# Why are Hoogsteen base pairs energetically disfavored in A-RNA compared to B-DNA?

**DOI:** 10.1093/nar/gky885

**Published:** 2018-10-04

**Authors:** Atul Rangadurai, Huiqing Zhou, Dawn K Merriman, Nathalie Meiser, Bei Liu, Honglue Shi, Eric S Szymanski, Hashim M Al-Hashimi

**Affiliations:** 1 Department of Biochemistry, Duke University School of Medicine, Durham, NC, USA; 2 Department of Chemistry, Duke University, Durham, NC, USA; 3 Goethe University, Institute for Organic Chemistry and Chemical Biology, Frankfurt am Main, Germany

## Abstract

A(*syn*)-U/T and G(*syn*)-C^+^ Hoogsteen (HG) base pairs (bps) are energetically more disfavored relative to Watson–Crick (WC) bps in A-RNA as compared to B-DNA by >1 kcal/mol for reasons that are not fully understood. Here, we used NMR spectroscopy, optical melting experiments, molecular dynamics simulations and modified nucleotides to identify factors that contribute to this destabilization of HG bps in A-RNA. Removing the 2′-hydroxyl at single purine nucleotides in A-RNA duplexes did not stabilize HG bps relative to WC. In contrast, loosening the A-form geometry using a bulge in A-RNA reduced the energy cost of forming HG bps at the flanking sites to B-DNA levels. A structural and thermodynamic analysis of purine-purine HG mismatches reveals that compared to B-DNA, the A-form geometry disfavors *syn* purines by 1.5–4 kcal/mol due to sugar-backbone rearrangements needed to sterically accommodate the *syn* base. Based on MD simulations, an additional penalty of 3–4 kcal/mol applies for purine-pyrimidine HG bps due to the higher energetic cost associated with moving the bases to form hydrogen bonds in A-RNA versus B-DNA. These results provide insights into a fundamental difference between A-RNA and B-DNA duplexes with important implications for how they respond to damage and post-transcriptional modifications.

## INTRODUCTION

**Figure 1.**
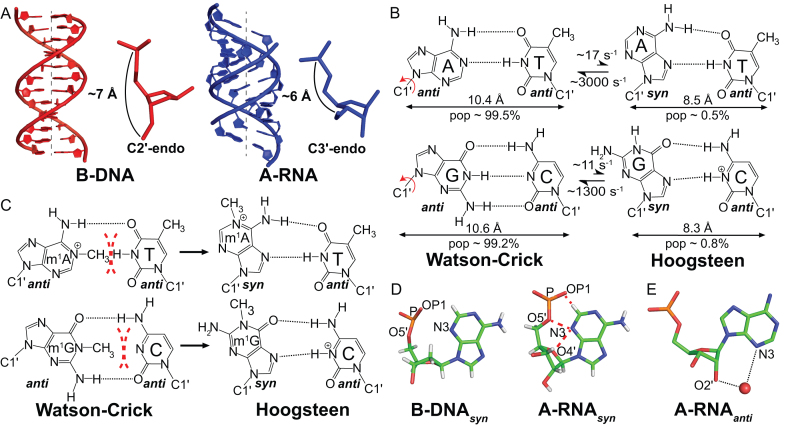
Hoogsteen base pairs in DNA and RNA. (**A**) Structural comparison of B-form DNA and A-form RNA. (**B**) WC bps in B-DNA exist in dynamic equilibrium with HG bps. Rates and populations were obtained using NMR relaxation dispersion methods as described previously (18). (**C**) *N*^1^-methylated purines disrupt WC bps via steric clashes (red dashes) and loss of hydrogen bonding interactions. They are accommodated as HG bps in B-DNA. (**D**) *Syn* purine bases clash (red dashes) with the sugar and backbone in A-RNA but not in B-DNA. (**E**) Water mediated interactions between the 2′-hydroxyl and the N3 atom of the purine base may stabilize purine bases in the *anti* conformation in A-RNA (PDB ID: 315D).

A-form RNA (A-RNA) and B-form DNA (B-DNA) double helices have several important differences (Figure 1A). In B-DNA, the deoxyribose moiety is flexible and exists in dynamic equilibrium between major C2′-endo and minor C3′-endo sugar pucker conformations (Figure 1A) (1,2). Steric clashes between the 2′-hydroxyl of a C2′-endo ribose sugar and the backbone, in conjunction with electronic effects of the 2′-hydroxyl preclude the formation of a corresponding B-form RNA double helix (3–5). Rather, the ribose primarily adopts the C3′-endo conformation in A-RNA. This brings into proximity adjacent nucleotides thus shortening the double helix and moving bps away from the helical axis (6) (Figure 1A). The resulting A-RNA double helix is also more rigid than its B-DNA counterpart (7–9). These differences between A-RNA and B-DNA have important biological implications for their recognition by proteins (10,11) and ligands (12), the templated processes of replication, transcription and translation, the consequences of ribonucleotide and deoxyribonucleotide misincorporation (13,14), and the impact of damage (15) and chemical modifications.

We recently reported markedly different propensities between B-DNA and A-RNA duplexes to form G(*syn*)-C^+^ and A(*syn*)-T/U Hoogsteen (HG) base pairs (bps) (15). A HG bp is created by rotating the purine base in a Watson–Crick (WC) bp ∼180° about the glycosidic bond to adopt a *syn* (0° < χ < 90°, where χ is the glycosidic O4′–C1′–N9–C4 torsion angle for purines) rather than an *anti* (−180° < χ < −90°) conformation, followed by translation of the two bases by ∼2.0 Å, creating a unique set of hydrogen bonds (h-bonds) (Figure 1B) (16). In B-DNA, the free energy associated with converting G–C or A–T WC bps into their HG counterparts (ΔG_HG-WC_) is estimated to be ∼2–4 kcal/mol based on NMR relaxation dispersion (RD) and optical melting experiments (15,17,18). In contrast, the free energy cost for forming HG bps in A-RNA (∼4–7 kcal/mol) is higher by ∼1–5 kcal/mol (15).

The difference in the propensities of A-RNA and B-DNA to form HG bps has important biological implications. Chemically modified nucleotides such as *N*^1^-methyladenonisine (m^1^A) and *N*^1^-methylguanosine (m^1^G), which are impaired from forming WC bps (Figure 1C), occur as a form of damage in both DNA (19) and RNA (20), and as post-transcriptional modifications in RNA (21). B-DNA is able to absorb this form of alkylation damage through the formation of HG bps (Figure 1C) (17,22), which can in turn be recognized by damage repair enzymes (19,23), safeguarding the integrity of the genome. In contrast, because of the instability of HG bps in A-RNA, these modifications disrupt base pairing all together (15). This enables m^1^A and m^1^G to serve unique functional roles as post-transcriptional modifications that direct the proper folding (24,25) and aminoacylation of tRNA (26), and prevent frameshifting errors during translation (27). A similar mechanism has also been proposed (15) to be responsible for the ability of m^1^A to increase translation (28,29) by destabilizing the 5′ UTR of mRNA. These functions would not be realized if m^1^A and m^1^G could form HG bps in A-RNA. A deep understanding regarding the factors that dictate the differences in HG bp stability relative to WC in B-DNA versus A-RNA is important for understanding the basis of these biological processes and may also provide guidelines that could help identify other processes in which the suppression or enhancement of HG bps is important.

Based on computational modeling and a survey of crystal structures, we proposed previously that HG bps are energetically more disfavored in A-RNA as compared to B-DNA due to steric clashes between the *syn* base and ribose sugar (N3–O4′), and the phosphate backbone (N3–O5′, N3-OP1) (15) (Figure 1D). These clashes arise due to the C3′-endo sugar pucker in RNA, which brings the *syn* purine base and phosphate group closer together. A similar mechanism has also been proposed to be responsible for the tendency of C3′-endo sugars to disfavour the *syn* conformation of the purine base in isolated NTPs (30–32). In contrast to RNA, base-backbone steric clashes are not observed for *syn* purines in B-DNA owing to its altered C2′-endo sugar pucker (Figure 1D). Although this simple steric model is appealing, there is reason to believe that it does not fully capture all the factors contributing to HG bp instability in A-RNA.

Based on X-ray crystallography (33–35) and solution state NMR spectroscopy (36–38), purine-purine mismatches such as G(*syn*)-G and G(*syn*)-A^+^/G-A(*syn*) adopt HG bps in both A-RNA and B-DNA duplexes. This indicates that any steric clashes with the *syn* purine base can be resolved through conformational adjustments. Moreover, the free energy associated with these adjustments likely does not exceed the energetic cost of base opening (39), otherwise well-formed purine-purine HG bps would not be observed in solution. This raises the possibility that there are other factors contributing to the instability of A(*syn*)-U and G(*syn*)-C^+^ HG bps in A-RNA. In addition, other contributions involving the 2′-hydroxyl need to be re-assessed because X-ray crystallography (40,41), NMR spectroscopy (42,43) and computational simulations (44) provide evidence for water mediated h-bonds between the 2′-hydroxyl and the base atoms in the minor groove of RNA (Figure 1E) (45). These h-bonds with WC bps would be disrupted when forming a *syn* base and could also account, at least in part, for the greater instability of HG bps relative to WC bps in A-RNA versus B-DNA. Finally, differences in stacking interactions of the *syn* purine base and the strengths of h-bonds it forms could also contribute to the differences in the propensities of RNA and DNA duplexes to form HG bps.

Here, we used NMR spectroscopy, melting experiments, molecular dynamics (MD) simulations as well as chemically modified nucleotides to dissect the origins of HG bp instability in A-RNA. Our results indicate that the A-form geometry destabilizes purine-pyrimidine and purine-purine HG bps due to the energetic cost associated with changing the sugar-backbone conformation to accommodate the *syn* purine base (by 1.5–4 kcal/mol as compared to DNA). Furthermore, an additional penalty of 3–4 kcal/mol as compared to B-DNA applies for the formation of purine-pyrimidine HG bps in A-RNA that is associated with the translation of bases into hydrogen bonding proximity. These results provide deeper insights into a fundamental difference between RNA and DNA duplexes and have important implications for how A-RNA and B-DNA respond to damage and post-transcriptional modifications.

## MATERIALS AND METHODS

### Sample preparation

Unmodified DNA and m^1^rG containing oligonucleotides: All unmodified DNA oligonucleotides were purchased from Integrated DNA Technologies with standard desalting purification, while all m^1^rG containing single stranded oligonucleotides (A_6_-DNA^m1rG10^, A_6_-RNA^m1rG10^, HIV-2 TAR^m1rG26^, A_2_-RNA^m1rG10^, gcRNA^m1rG4^) were purchased from GE Healthcare Dharmacon with HPLC purification.

m^1^dA and m^1^rA containing DNA and RNA oligonucleotides: The A_6_-DNA^m1rA16^ single-strand was purchased from Yale Keck Oligonucleotide Synthesis Facility with Glen-Pak RNA cartridge purification while the A_6_-DNA^m1dA16^ single-strand was purchased from Midland Certified Reagents with reverse-phase HPLC purification. The A_6_-RNA^m1rA16^ and A_6_-RNA^m1dA16^ single-stranded oligonucleotides were synthesized in-house using a MerMade six oligo synthesizer. Ultramild TBDMS RNA amidites (Pac-rA, Ac-rC, iPr-Pac-rG, rU, Glen Research Corporation), m^1^A phosphoramidites (Glen Research Corporation) and Ultramild Cap A solution were used with a coupling time of 12 min, with the final DMT group being cleaved during the synthesis. The RNA oligonucleotides were then cleaved and deprotected after support removal using a 2 ml solution of 2 M ammonia in methanol for 24 h at room temperature. The solutions were then centrifuged and the supernatant dried under airflow. The resulting oligonucleotide crystals were dissolved in 100 μl DMSO and 125 μl TEA.3HF, and heated at 65°C for 2.5 h for 2′-*O* deprotection. The oligonucleotides were then ethanol precipitated and dissolved in a formamide based loading dye for purification using PAGE. Gel bands corresponding to the pure product were identified by UV-shadowing and subject to electroelution (Whatman, GE Healthcare) followed by ethanol precipitation.

RNA oligonucleotides used for comparison with m^1^rA/m^1^dA containing RNA duplexes in optical melting experiments: All the unmethylated RNA single strands used to prepare A_6_-RNA, A_6_-RNA^dA16^, A_6_-RNA^m1dA16^ and A_6_-RNA^m1rA16^ duplexes that were compared with the m^1^rA/m^1^dA containing RNA duplexes in the optical melting measurements were synthesized in-house using a MerMade 6 oligo synthesizer. In particular, acetyl protected TBDMS RNA amidites (Chemgenes) and standard DNA phosphoramidites (n-bz dA, Chemgenes) were used with a coupling time of 6 min for RNA and 1 min for DNA, with the final 5′-DMT group removed during synthesis. The oligonucleotides were cleaved from the supports (1 μmol) using ∼1 ml of AMA (1:1 ratio of ammonium hydroxide and methylamine) for 30 min and deprotected at room temperature for 2 hrs. All subsequent purification steps were similar to those used for the m^1^dA/m^1^rA containing RNA oligonucleotides.

m^1^dG and rNMP containing DNA oligonucleotides: The A_6_-DNA^m1dG10^ single strand was purchased from Midland Certified Reagents with cartridge purification. All other DNA single strands containing m^1^dG (gcDNA^m1dG4^ and A_2_-DNA^m1dG10^) or rNMP incorporations (A_6_-DNA^rG10^ and A_6_-DNA^rA16^), were synthesized in-house using a MerMade 6 oligo synthesizer. In particular acetyl protected TBDMS RNA phosphoramidites (Chemgenes) and standard DNA phosphoramidites (n-ibu-dG, bz-dA, ac-dC, dT, dmf-m^1^dG, Chemgenes) were used with a coupling time of 6 min (RNA) and 1 min (DNA), with the final 5′-DMT group retained during synthesis. The oligonucleotides were cleaved from the supports (1 μmol) using ∼1 ml of AMA (1:1 ratio of ammonium hydroxide and methylamine) for 30 min and deprotected at room temperature for 2 h. The m^1^dG containing DNA samples were then purified using Glen-Pak DNA cartridges and ethanol precipitated, while the rNMP containing samples were dried under airflow to obtain oligonucleotide crystals. They were then dissolved in 115 μl DMSO, 60 μl TEA and 75 μl TEA.3HF and heated at 65°C for 2.5 h for 2′-*O* deprotection. The samples were then neutralized using 1.75 ml of Glen-Pak RNA quenching buffer, loaded onto Glen-Pak RNA cartridges for purification and were subsequently ethanol precipitated.

Other RNA oligonucleotides: The remaining RNA single strands used for preparing A_6_-RNA, A_6_-RNA^dG10^, A_6_-RNA^m1dG10^, A_6_-RNA^m1rG10^, HIV-2 TAR, A_2_-RNA, A_2_-RNA^GG^, A_2_-RNA^m1rGG4^, gcRNA, gcRNA^GG^, gcRNA^m1rGG4^ samples were synthesized in-house using a MerMade 6 oligo synthesizer. In particular acetyl protected TBDMS RNA phosphoramidites (Chemgenes) and standard DNA phosphoramidites (*n*-ibu-dG, dmf-m^1^dG, Chemgenes) were used with a coupling time of 6 min (RNA) and 1 min (DNA), with the final 5′-DMT group retained during synthesis. The subsequent purification steps used were similar to those used for preparing the rNMP containing DNA oligonucleotides.

Sample annealing and buffer exchange: All single strands (after ethanol precipitation/purchase) were re-suspended in water. Duplex samples were prepared by mixing equimolar amounts of the constituent single strands and annealed by heating at 95°C for ∼5 min and cooling at room temperature for ∼1 h. All hairpin samples (HIV-2 TAR, HIV-2 TAR^m1rG26^) were prepared by diluting the re-suspended oligonucleotides to a concentration of ∼50 μM followed by heating at 95°C for ∼5 min and cooling on ice for ∼1 h. Extinction coefficients for all single and double stranded species were estimated using the ADTBIO oligo calculator (https://www.atdbio.com/tools/oligo-calculator). Following annealing, the samples were exchanged three times into the desired buffer using centrifugal concentrators (4 ml, Millipore Sigma).

Buffer preparation: Sodium phosphate buffers for NMR and optical melting measurements were prepared by the addition of equimolar solutions of sodium phosphate monobasic and dibasic salts, sodium chloride, EDTA and magnesium chloride to give final concentrations (unless mentioned otherwise) of 15 mM (phosphate), 25 mM, 0.1 mM and 3 mM, respectively. The pH of the buffers was adjusted by the addition of phosphoric acid, after which they were brought up to the desired volume, and filtered and stored for further usage. Potassium phosphate buffers for optical melting measurements were also prepared in an analogous manner using equimolar solutions of potassium phosphate monobasic and dibasic salts, and potassium chloride.

### NMR spectroscopy

NMR experiments were performed on an 800 MHz Varian DirectDrive2 spectrometer and a 700 MHz Bruker Avance 3 spectrometer equipped with triple-resonance HCN cryogenic probes. All experiments were conducted in pH 5.4, 25 mM NaCl and at 25°C unless stated otherwise. The NMR data was processed and analyzed with NMRpipe (46) and SPARKY (47). Resonances were assigned using 2D NOESY, TOCSY and DQF-COSY experiments along with SOFAST HMQC experiments for aromatic (48) and imino (49) spins. The TOCSY and DQF-COSY experiments were performed in D_2_O following sample lyophilization. The coupling constant *J*_H1′-H2′_ for rA16 and rG10 in A_6_-DNA^rA16^ and A_6_-DNA^rG10^, and ∑H1′ for dG10 in A_6_-RNA^dG10^ were measured along the direct dimension (ω_1_) of the DQF-COSY spectra after phasing of the relevant cross peak. Chemical shift perturbations (Δω) for a pair of resonances (C2-H2/C8-H8/C6-H6/C1′-H1′) belonging to a given residue were calculated using the following equation

\begin{equation*}\Delta \omega \ = \ \sqrt {{{\left( {\Delta {\omega _H}} \right)}^2} + \ {{\left( {\frac{{{\gamma _H}}}{{{\gamma _C}}}\ \Delta {\omega _C}} \right)}^2}} \end{equation*}

where Δ${\omega _H}$ and Δ${\omega _C}$ are the chemical shift perturbations in the hydrogen and carbon dimensions of a 2D CH HSQC spectrum, and ${\gamma _H}$ and ${\gamma _C}$ are the gyromagnetic ratios of hydrogen and carbon. A chemical shift perturbation (Δω) was considered to be significant when ≥ 0.4 ppm.

### Optical melting experiments

NMR samples were diluted using NMR buffer to a concentration of 3 μM and used for optical melting experiments (extinction coefficients for the modified duplexes were assumed to be the same as that for the unmodified ones. Modified bases are estimated to affect the extinction coefficient for the oligos used here by < 10% based on reference values in Basanta-Sanchez *et al*. (50)). Measurements were performed using a Perkin Elmer Lamba 25 UV–Vis spectrophotometer with a Peltier temperature control unit and a sample/blank volume of 400 μL. Samples were heated a rate of 1 °C/min with the absorbance at 260 nm (*A*_260_) being recorded every 0.5 min. The absorbance curves were then fit to obtain the thermodynamic parameters using an in-house Mathematica script and the following equations

\begin{equation*}{{\rm{A}}_{260}}{\rm{\ }} = {\rm{\ }}\left( {\left( {\left( {{{\rm{m}}_{{\rm{ds}}}}{\rm{*T}}} \right){\rm{\ }} + {\rm{\ }}{{\rm{b}}_{{\rm{ds}}}}} \right){\rm{*f}}} \right){\rm{\ }} + {\rm{\ }}\left( {\left( {\left( {{{\rm{m}}_{{\rm{ss}}}}{\rm{*T}}} \right){\rm{\ }} + {\rm{\ }}{{\rm{b}}_{{\rm{ss}}}}} \right){\rm{*}}\left( {1 - {\rm{f}}} \right)} \right)\end{equation*}

\begin{equation*}f\ = \frac{{\left( {1 + 4{{\rm{e}}^{\left( {1/{{\rm{T}}_{\rm{m}}} - 1/{\rm{T}}} \right){\rm{\Delta H}}/{\rm{R}}}}} \right) - {\rm{\ }}{{\left( {1 + 8{{\rm{e}}^{\left( {1/{{\rm{T}}_{\rm{m}}} - 1/{\rm{T}}} \right){\rm{\Delta H}}/{\rm{R}}}}} \right)}^{1/2}}}}{{4{{\rm{e}}^{\left( {1/{{\rm{T}}_{\rm{m}}} - 1/{\rm{T}}} \right){\rm{\Delta H}}/{\rm{R}}}}}}\end{equation*}

where ${\rm{\ }}{{\rm{m}}_{{\rm{ds}}}}$, ${{\rm{b}}_{{\rm{ds}}}}$, ${{\rm{m}}_{{\rm{ss}}}}$ and ${{\rm{b}}_{{\rm{ss}}}}$ are coefficients representing the temperature dependence of the extinction coefficients of the double and single stranded species, *T* is the temperature in Kelvin, *f* is the fraction of the double stranded species at a given temperature, *T*_m_ is the melting temperature in Kelvin, Δ*H* is the enthalpy of the melting transition in kcal/mol and *R* is the universal gas constant in kcal/mol/K.

The free energy of the melting transition was then obtained as follows

\begin{equation*}{\rm{S\ }} = {\rm{\Delta H}}/{{{T}}_{\rm{m}}} - {R\rm{ln}}\left( {{{{C}}_{{t}}}/2} \right)\,{\rm{and}}\,{{\Delta G}} = {{\Delta H}} - {{T\Delta S}}\end{equation*}

where *C*_*t*_ is the total concentration of the duplex species at the start of the measurement and Δ*S* and Δ*G* are the entropy and free energy of the melting process. While performing experiments on hairpin systems, the fraction of folded hairpin at a given temperature was defined by

\begin{equation*}{{f\ }} = {\rm{\ }}\frac{{{{\rm{e}}^{\left( {1/{{{T}}_{\rm{m}}} - 1/{{T}}} \right){{\Delta H}}/{{R}}}}}}{{1 + {\rm{\ }}{{\rm{e}}^{\left( {1/{{{T}}_{\rm{m}}} - 1/{{T}}} \right){{\Delta H}}/{{R}}}}}}\end{equation*}

The *T*_m_ and Δ*H* from fitting the absorbance curve were then used to get the free energy as follows

\begin{equation*}{{\Delta S\ }} = {{\ \Delta H}}/{{{T}}_{\rm{m}}}\,{\rm{and}}\,{{\Delta G\ }} = {{\ \Delta H}} - {{T\Delta S}}\end{equation*}

Errors in the thermodynamic measurements (one standard deviation) were estimated by performing the experiments in triplicate.

### MD simulations

All MD simulations were performed using the ff99 AMBER force field (51) with bsc0 corrections for DNA (52) and χ_OL3_ corrections (53) for RNA, using periodic boundary conditions as implemented in the AMBER MD package (54). Starting structures for A_6_-DNA with the A16-T9 base pair in a WC/HG/HG* conformation were obtained from the NMR structures of A_6_-DNA (PDB ID: 5UZF) and A_6_-DNA^m1dA16^ (PDB ID: 5UZI) (55). HG* (56) refers to a A(*syn*)-T bp conformation in which the *syn* adenine is not hydrogen bonded to the thymine as the C1′–C1′ distance across the bp is not constricted. The deposited 5UZF structure was used as is, to model the WC conformation of the A16-T9 bp. The HG conformation was modeled by removing the N^1^-methyl group from 5UZI while the HG* conformation was modeled by flipping the A16 base in 5UZF 180° about the glycosidic bond. The starting structure for A_2_-DNA with the A16-T9 bp in a WC conformation was obtained from PDB ID 5UZD (NMR structure of A_2_-DNA) while that in a HG* conformation was derived from 5UZD by flipping the A16 base by 180° about the glycosidic bond. The starting structure for A_2_-DNA with a HG conformation of the A16-T9 bp was obtained from Sathyamoorthy *et al*. (55). Starting structures for the A_6_ and A_2_ RNA systems with the A16-U9 bp in a WC/HG/HG* conformation were generated by constructing idealized helices using 3DNA (57). The HG conformation was modeled by superimposing (using base heavy atoms) the m^1^A-T HG bp from 5UZI (after removing the thymine and *N*^1^-methyl groups) onto the A16-U9 bp (in the idealized structure, with A16 in the *syn* conformation) and replacing the atoms of both bases with those in the superimposed HG bp. The HG* conformation was modeled by flipping the base moiety of A16 in the idealized structure about the glycosidic bond by 180°. Starting structures for A_6_-DNA and A_6_-RNA with the G10-C15 bp in a HG* conformation were generating by rotating G10 180° about the glycosidic bond in 5UZF and an idealized helix respectively. The HG conformation for the G10-C15^+^ bp was modeled in a manner similar to that for A16-U9 bp in A_6_-RNA for both systems, with the reference G(*syn*)-C^+^ HG bp taken from PDB ID 1XVK (bp 1:8). Parameters for protonated cytosine (for the HG and HG* starting geometries of the G10-C15 bp) were obtained from Goh *et al*. (58). Starting structures for the A_6_ and A_2_ DNA and RNA duplexes containing a G-G mismatch at position 10:15 were built by constructing idealized helices using 3DNA (57). In particular, the base atoms of the G10(*syn*)-G15(*anti*) bp (obtained by replacing C15 in the idealized structure with an *anti* guanine) were replaced with those of a superimposed (using the base heavy atoms of the *anti* guanine) G-G mismatch from PDB ID 1D80 (bp 9:16). All helices were then solvated using a truncated octahedral box of SPC/E (59) water molecules, with box size chosen such that the boundary was at least 10 Å away from any of the DNA atoms. Na^+^ ions treated using the Joung-Cheatham parameters (60) were then added to neutralize the charge of the system. The system was then energy minimized in two stages with the solute (except for the sugar moiety of the A16-U9/G10-C15^+^ bps in the HG conformation, and the sugar atoms of the G-G mismatch) being fixed (with a restraint of 500 kcal/mol/Å^2^) during the first stage. This was followed by gradual heating of the system using the Berendsen thermostat (61) to 298 K under constant volume conditions for 100 ps with harmonic restraints on the solute (10 kcal/mol/Å^2^). The system was then allowed to equilibrate for 1 ns under constant pressure (1 bar, using the Berendsen barostat, τ = 2 ps) and temperature (at 298 K, using Langevin dynamics, γ = 3 ps^−1^) conditions. A non-bonded cutoff of 9 Å was used for treating short range non-bonded interactions while the Particle Mesh Ewald method (62) was used to treat long range electrostatic interactions. Covalent bonds involving hydrogen were constrained using the SHAKE algorithm (63) to enable the use of a 2 fs timestep. Simulations of A_6_-DNA with HG and HG* starting geometries of the A16(*syn*)-T9 bp, and a G10(*syn*)-G15 mismatch were also performed using the ff99 AMBER force field (51) with the recently developed parmbsc1 (64) and OL15 (65) corrections for DNA, using the protocol described above. Production runs of length 1 μs were obtained for all the simulations. The settings used for the production runs were identical to those used during equilibration. A set of evenly (5 ps) spaced snapshots was used for subsequent analysis using the CPPTRAJ suite (66) of programs. Visual examination of the MD trajectories revealed the absence of terminal end fraying artifacts near the base pair of interest in all simulations apart from that of A_6_-DNA containing a G10(*syn*)-G15(*anti*) mismatch (bsc0 force field, after 650 ns). The terminal base pairs in this simulation were seen to interact with the mismatch via insertion in both the minor and major grooves. However, this did not affect the stability of the G-G mismatch (Supplementary Figure S9D). Furthermore, examination of the RMSD of the heavy atoms (excluding the terminal residues) of DNA/RNA during the production runs for all simulations suggests that they are converged (Supplementary Figure S7).

### Survey of crystal structures with purine-purine mismatches

All crystal structures containing nucleic acids with a resolution < 3 Å as of 27 April 2017 were downloaded from the Protein Data Bank (PDB) (67) and analyzed for the presence of purine-purine mismatches formed by the canonical bases and their modified derivatives, that are flanked by two WC bps on both sides to mimic a duplex like environment, using an in-house Python script. The mismatches were then classified based on the χ angle of the constituent bases to obtain *syn-anti*/HG conformations for all mismatch types (A-A/A-G/G-G). Two structures of MutS bound to DNA containing purine-purine mismatches (1OH6 and 1OH7) were manually excluded owing to the distorted/open geometry of the mismatch caused by the intercalation of aromatic amino acids from MutS in the DNA minor groove, as they are unlikely to be representative of the accommodation of a mismatch in a duplex context. A total of 69 purine-purine HG bps belonging to 37 distinct structures were identified out of a total of 5906 deposited structures in the PDB containing nucleic acids. Additional statistics obtained from the survey along with the identified HG purine-purine mismatches are shown in Tables S3 and S4 in the supporting material. The torsion angles of the mismatched bps were compared with a set of unmodified WC bps from free (not bound to proteins/ligands) DNA/RNA structures placed in a similar structural context, with B/A form helical geometry as determined using DSSR (68). Purine-purine HG bps were also compared with a set of isolated purine-pyrimidine HG bps in DNA (flanked by WC bps on both sides) identified earlier (56). No crystal structures of purine-pyrimidine HG bps in duplex environments in RNA were found, in line with a previous study (15).

### Calculation of changes in stacking interactions accompanying the formation of purine-purine mismatches

Triplets of bps containing the HG purine-purine mismatches in Supplementary Table S4 along with their neighboring bps were extracted from the respective PDB files. Modified bases such as inosine, 5-bromo uridine, 8-bromo guanosine were replaced with the canonical bases (A/T/U/G/C) by adding/removing the extra atoms. For each mismatched triplet, a corresponding WC base paired triplet was created by constructing an idealized B/A-form helix with 3-DNA (57), by using the sequence of the mismatched strand containing the *syn* base. For example, the WC base paired triplet corresponding to the mismatched sequence 5′-TA(*syn*)G-3′/5′-CG(*anti*)A-3′ would be 5′-TAG-3′/5′-CTA-3′. The stacking interactions in the triplet of matched and mismatched bps were calculated by computing the area of base overlap using the *analyze* utility of the 3-DNA suite (57). The change in overlap area between the mismatched and matched triplets is computed with the inclusion of exocyclic groups in Supplementary Figure S6F and Supplementary Table S5 in the supporting material.

### Computation of thermodynamic parameters for mismatch formation

Thermodynamic parameters for G-G and T-T/U-U mismatch formation (relative to a G-C bp) were computed using MELTING 5.0 (69) for all possible sequence contexts surrounding the mismatch for both DNA and RNA. Default options for nearest neighbor thermodynamic parameters and ion correction terms were used along with a sodium ion concentration of 150 mM. The energetic terms for helix initiation and symmetry were set to zero, in order to mimic the placement of a mismatch within the context of a non-palindromic duplex.

## RESULTS

### Removing the 2′-hydroxyl at a purine nucleotide does not rescue HG bp formation in A-RNA

**Figure 2.**
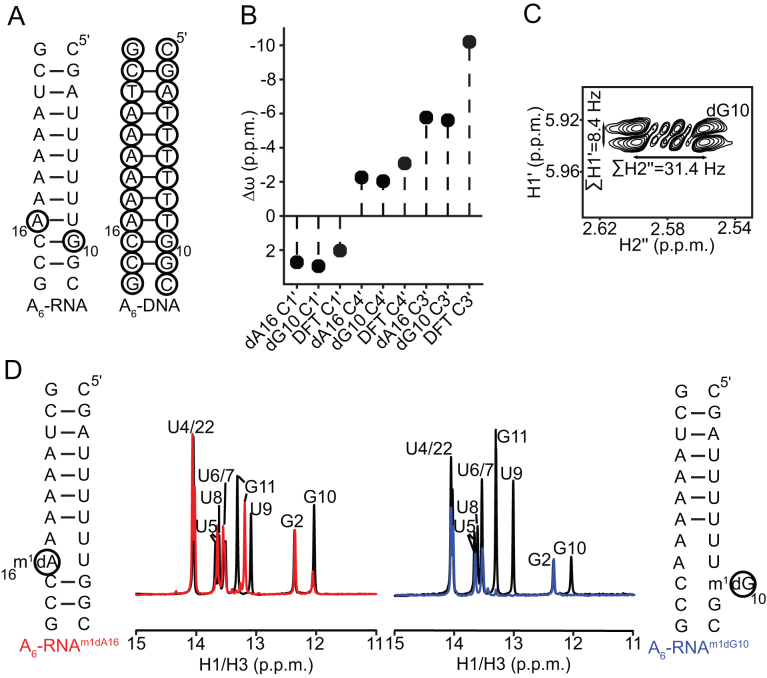
Removal of the 2′-hydroxyl at a purine nucleotide minimally affects the sugar geometry in A_6_-RNA and does not rescue HG bp formation. (**A**) Secondary structure of A_6_-RNA with the sites of dNMP incorporation indicated using black circles, and A_6_-DNA. (**B**) Chemical shift perturbations of the sugar resonances of the dNMPs in A_6_-RNA relative to the corresponding dNMP residues in A_6_-DNA. Also shown are DFT predicted (70) chemical shift perturbations for the transition of deoxyribonucleosides from a C2′-endo (χ = -100°) to a C3′-endo conformation (χ = −160°). The discrepancy with the expected change in the C3′ chemical shift is likely due to the exclusion of the 3′-phosphate group from the chemical shift calculations (70). (**C**) DQF-COSY spectrum of A_6_-RNA^dG10^ showing the sums of the scalar couplings (∑H1′ = *J*_H1′-H2′_ + *J*_H1′-H2″_ and ∑H2″ = *J*_H1′-H2″_ + *J*_H2′-H2″_ + *J*_H2″-H3′_) for the H1′ and H2″ protons at the dG10 residue. The corresponding scalar couplings could not be measured for dA16 in A_6_-RNA^dA16^ due to severe overlap of the H1′-H2′ and H1′-H2″ cross peaks in the DQF-COSY spectra. Ranges of ∑H1′ and ∑H2″ for C3′-endo and C2′ -endo deoxyribose are 8–11 Hz and 30–33 Hz, and 15–16 Hz and 19–21 Hz respectively (72). (**D**) Comparison of ^1^H 1D spectra of the imino region of A_6_-RNA^m1dA16^ (red) and A_6_-RNA^m1dG10^ (blue), with the spectra for A_6_-RNA^dA16^ and A_6_-RNA^dG10^ (black). All NMR spectra were collected in pH 5.4, 25 mM NaCl and at 25°C.

Based on the steric model (Figure 1D) (15) HG bps are disfavored in A-RNA as compared to B-DNA due to steric clashes that do not involve the 2′-hydroxyl group. Thus, we predict that removal of the 2′-hydroxyl at a purine nucleotide in RNA should not result in a resurgence of stably formed HG bps on *N*^1^-methylation, as long as the local conformation remains A-form. On the contrary, if HG bps are destabilized relative to WC bps in RNA solely due to favorable water mediated (Figure 1E) (45) or other interactions of the hydroxyl with the WC bp, then we predict that its removal should result in the occurrence of stable HG bps on *N*^1^-methylation. To test these predictions, we examined the consequence of removing the 2′-hydroxyl at a purine nucleotide (rA16 or rG10) in the A_6_-RNA duplex (Figure 2A).

Comparison of 2D HSQC NMR spectra for dA16 or dG10 substituted A_6_-RNA duplexes with their unmodified counterparts indicates that deoxyribose substitution does not alter the global A-form conformation and results in small chemical shift perturbations in and around the site of substitution (Supplementary Figures S1A and S1B). The aromatic carbon and proton chemical shifts of the dNMP residues are consistent with a helical A-form RNA conformation (Supplementary Figures S1A and S1B). In addition, the sugar carbon (C1′, C3′ and C4′) chemical shifts (70,71) and scalar coupling constants (∑H1′ = *J*_H1′-H2′_ + *J*_H1′-H2″_ and ∑H2″ = *J*_H1′-H2″_ + *J*_H2′-H2″_ + *J*_H2″-H3′_) (2,72) indicate that the dNMP residue adopts a C3′-endo sugar pucker as expected for an A-form conformation (Figure 2B and C).

As the conformation remains A-form on hydroxyl removal, HG bp formation should still be disfavored at the dNMP residue based on the steric model. Indeed, ^1^H 1D spectra of the imino region of the A_6_-RNA duplexes containing m^1^dA and m^1^dG show an absence of characteristic hydrogen bonding signatures corresponding to HG bp formation (17) and a lack of base pairing near the site of *N*^1^-methylation (Figure 2D). Furthermore, characteristic NMR signatures of HG bp formation such as downfield shifted C8 resonances and strong intra-nucleotide H1′-H8 cross peaks corresponding to the *syn* purine are not observed (Supplementary Figure S2A-C). This indicates that the duplexes adopt partially melted conformations just like their m^1^rA and m^1^rG counterparts (15), although we cannot rule out that hydrogen bonded HG bps are not formed transiently.

The above experiments suggest that the effects of *N*^1^-methylation on A_6_-RNA are independent of the presence/absence of the 2′-hydroxyl. This was independently validated using optical melting measurements on modified A_6_-RNA duplexes. The destabilization due to the incorporation of m^1^A or m^1^G (ΔG_N1methyl-WC_), computed as the difference in free energies of melting between the *N*^1^-methylated duplex and its unmethylated counterpart, was seen to be minimally affected (by 0.1–0.5 kcal/mol) on removal of the 2′-hydroxyl under a variety of experimental conditions such as low (Supplementary Figure S2D) and moderate salt (Supplementary Figure S2E), presence of magnesium (Supplementary Figure S2F), neutral pH (Supplementary Figure S2G) and presence of potassium (Supplementary Figure S2H). Taken together, the above results argue against the loss of stabilizing interactions with the purine 2′-hydroxyl as being the primary source of instability of HG bps relative to WC bps in A-RNA compared to B-DNA, and are in accordance with the steric model.

### Adding the 2′-hydroxyl at a purine nucleotide does not significantly destabilize HG bps relative to WC in B-DNA

**Figure 3.**
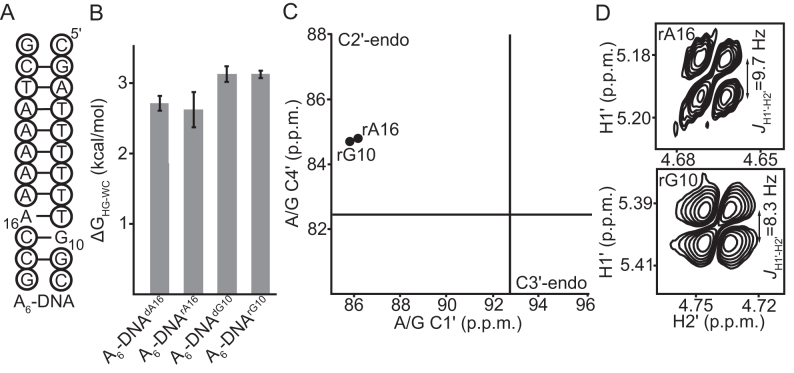
Addition of a 2′-hydroxyl to purine nucleotides in A_6_-DNA does not affect sugar geometry and minimally impacts HG bp formation. (**A**) Secondary structure of A_6_-DNA with the dNMP residues indicated using black circles. The sites of rNMP incorporation are G10 and A16. (**B**) The energetic cost of accommodating HG bps relative to WC (Δ*G*_HG-WC_) in DNA estimated from melting experiments on A_6_-DNA constructs with and without *N*^1^-methylated purine nucleotides at pH 5.4, 25 mM NaCl and 25°C. Errors in ΔG_HG-WC_ were obtained by propagating the errors from triplicate measurements (see ‘Materials and Methods’). (**C**) C1′ and C4′ chemical shifts of the rNMPs in A_6_-DNA fall in the C2′-endo quadrant of a C1′/C4′ correlation plot (77). Black lines denote the average chemical shits of helical A/G residues in RNA as determined from a survey of the BMRB (77). (D) DQF-COSY spectra showing the H1′-H2′ coupling constants of the rNMP sugars in A_6_-DNA (pH 5.4, 25 mM NaCl and 25°C). The *J*_H1′-H2′_ coupling is 8–10 Hz and 0–2 Hz for a C2′-endo and C3′-endo ribose sugar respectively.

We previously performed the inverse experiment and examined the consequence of introducing a 2′-hydroxyl group at a purine nucleotide in B-DNA on the stability of HG bps relative to WC. NMR RD measurements on rNMP (rG10 and rA16) substituted A_6_-DNA duplexes (Figure 3A) revealed that addition of the 2′-hydroxyl group slightly increases the energetic cost of forming HG bps relative to WC (Δ*G*_HG-WC_) by 0.2–0.3 kcal/mol (15). We corroborated these findings using optical melting experiments on the rNMP substituted A_6_-DNA duplexes with and without *N*^1^-methylation at G10 and A16 (Figure 3B). These experiments yielded changes in Δ*G*_HG-WC_ on addition of the 2′-hydroxyl that are <0.1 kcal/mol (Figure 3B). Addition of the hydroxyl to A_6_-DNA resulted in destabilization of both the WC and HG bps by 0.2–0.9 kcal/mol (Supplementary Figure S3, Supplementary Table S1). These results can help explain the faster rate of WC/HG exchange observed in the prior RD measurements on rNMP substituted A_6_-DNA duplexes (15). In particular, this arises because addition of the 2′-hydroxyl destabilizes both the WC and HG states while having a smaller destabilizing effect on the transition state (Supplementary Figure S3).

The above results are consistent with the steric model provided the local conformation at the rNMP residue remains B-form. Testing this prediction was important given that prior NMR (73,74) and X-ray crystallography (75,76) studies showed that single ribonucleotides in B-DNA can adopt a local A-form conformation in sequence contexts that differ from A_6_-DNA. However, based on NMR chemical shifts (70,77), and sugar coupling constants (*J*_H1′-H2′_) (78,79) we find that in the case of A_6_-DNA, the rNMPs adopt a C2′-endo pucker, as expected if the local conformation remains B-form on hydroxyl addition (Figure 3C and D). Taken together, these results argue against the direct involvement of interactions involving a single 2′-hydroxyl group in determining the HG bp forming propensity of A-RNA and B-DNA, consistent with the steric model.

### Loosening the A-form geometry stabilizes a G(*syn*)-C^+^ HG bp in HIV-2 TAR RNA

**Figure 4.**
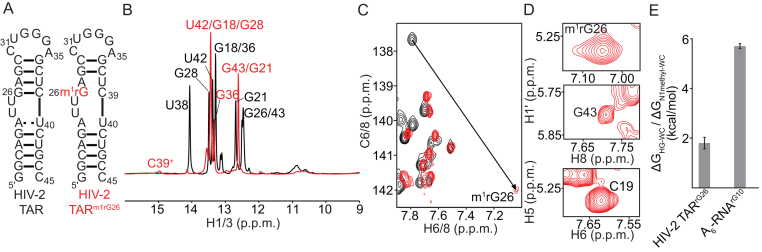
Relaxing the geometric restraints of the A-form stabilizes HG bps relative to WC in HIV-2 TAR RNA. (**A**) Secondary structure of HIV-2 TAR and HIV-2 TAR^m1rG26^. (**B**) Overlay of ^1^H 1D SOFAST imino spectra of HIV-2 TAR (black) and HIV-2 TAR^m1rG26^ (red) showing a downfield shifted imino peak corresponding to protonated C39^+^ that is hydrogen bonded to the *syn* m^1^rG26. (**C**) Overlay of the aromatic 2D CH HSQC spectra of HIV-2 TAR (black) and HIV-2 TAR^m1rG26^ (red) showing a downfield shift of G26-C8 by ∼4 ppm on *N*^1^-methylation. (**D**) The medium and weak intra-nucleotide H1′-H8 NOE cross peaks for m^1^rG26 and G43 in HIV-2 TAR^m1rG26^ that are indicative of a *syn* and *anti* conformation of the purine base respectively, compared to the H5-H6 cross peak of a reference cytosine C19. (**E**) Energetic cost of forming HG bps relative to WC (Δ*G*_HG-WC_) at the G26-C39 bp in HIV-2 TAR compared to the energetic cost of *N*^1^-methylation (Δ*G*_N1methyl-WC_) at the G10-C15 bp in A_6_-RNA (pH 5.4, 25 mM NaCl and 25°C). Errors in Δ*G*_HG-WC_ were obtained by propagating the errors from triplicate measurements (see Materials and Methods). All NMR spectra were collected at pH 5.8, 25 mM NaCl and 25°C.

According to the steric model, loosening the A-form geometry should help resolve steric clashes involving the *syn* base and reduce ΔG_HG-WC_ in RNA. We tested this prediction by examining the HG bp forming propensity of a G26-C39 WC bp that is adjacent to a dinucleotide bulge in human immunodeficiency virus type 2 (HIV-2) transactivation response element (TAR) RNA (Figure 4A). Here, the bulge is expected to relax the conformational restraints of the A-form helix. In prior studies, we showed that m^1^G or m^1^A do not form detectable HG bps in A-RNA duplexes under a wide variety of conditions (15). In contrast, NMR analysis of HIV-2 TAR RNA containing m^1^rG26 shows that it forms an m^1^G26(*syn*)-C39^+^ HG bp. In particular, we observe a downfield shift of the m^1^rG26-C8 resonance (by ∼4 ppm) (Figure 4C) (15,70) and a strong intra-nucleotide H1′-H8 NOE cross peak (Figure 4D) which indicate that the m^1^rG26 base adopts a *syn* conformation. Furthermore, we also observe a downfield shifted imino proton at ∼15 ppm corresponding to C39-H3^+^ that is hydrogen bonded to the *syn* m^1^rG26 (Figure 4B) (80,81) and downfield shifted amino protons belonging to protonated C39 (Supplementary Figure S4C) (17,80). However, the m^1^rG26-C1′ resonance is not downfield shifted (Supplementary Figure S4A) as would be expected for a HG bp in DNA (17), presumably due to the adoption of an altered sugar pucker/χ angle in the m^1^rG26(*syn*)-C39^+^ HG bp (70,77,82,83).

Optical melting experiments on m^1^rG26 modified, and unmodified HIV-2 TAR yielded a ΔG_HG-WC_ value of 1.8 ± 0.5 kcal/mol for the G26-C39 bp (Figure 4E). This is comparable to ΔG_HG-WC_ values measured in B-DNA (1.8–3.4 kcal/mol) (15) and is much lower than the Δ*G*_N1methyl-WC_ value (5.7 ± 0.1 kcal/mol) measured in A_6_-RNA for the G10-C15 bp (Figure 4E). Therefore, loosening the A-form geometry leads to stabilization of HG bps relative to WC bps, in line with the steric model.

### Accommodating a *syn* purine base is energetically more costly in A-RNA compared to B-DNA

**Figure 5.**
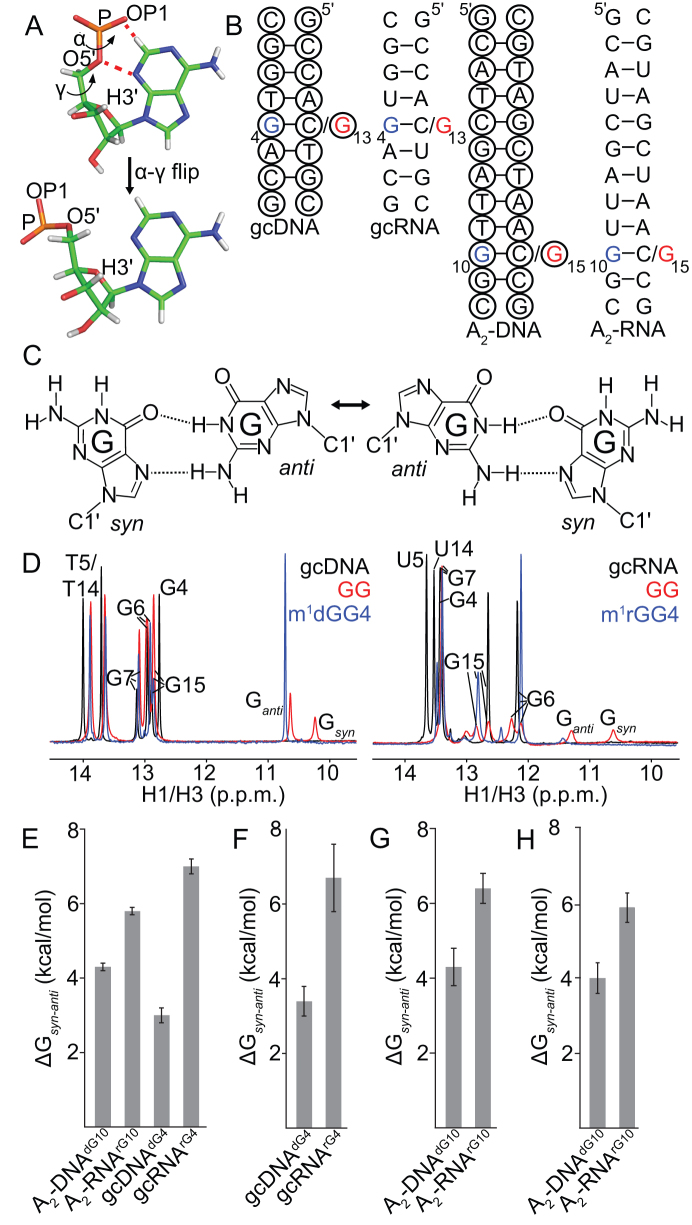
The energetic cost of accommodating the *syn* base contributes to HG bp instability in RNA. (**A**) Conformational changes in the α and γ backbone torsions involved in accommodating the steric clash (red dashes) with the *syn* purine in purine-purine HG bps in A-RNA (PDB ID: 4YN6). (**B**) Secondary structures of DNA and RNA constructs (gc and A_2_ sequence contexts) containing G–G mismatches. The C residue that is mutated to a G is indicated in red, while the position of m^1^G substitution (in the context of a G–G mismatch) is denoted in blue. The dNMP residues are indicated using black circles. (**C**) Dynamic exchange of G–G mismatches between *syn–anti* and *anti–syn* HG geometries in duplex DNA and RNA. (**D**) ^1^H 1D NMR spectra of the imino region of gcDNA and gcRNA duplexes with G–G mismatches (pH 5.4, 25 mM NaCl and 10°C). For gcRNA^GG^, we observe multiple imino peaks corresponding to the bps neighboring the mismatch that are in slow exchange on the chemical shift timescale. (E–G) The energetic cost of accommodating a *syn* base in RNA and DNA (ΔG*_syn_*_-_*_anti_*) estimated from optical melting measurements of constructs with m^1^G-G mismatches and C-G bps at (**E**) moderate salt (pH 5.4, 150 mM NaCl and 25°C), (**F**) in the presence of magnesium (pH 5.4, 150 mM NaCl, 3 mM MgCl_2_ and 25°C), and (**G**) low salt conditions (pH 5.4, 25 mM NaCl and 25°C). Estimates of ΔG*_syn_*_-_*_anti_* for A_2_-DNA and A_2_-RNA that were obtained by comparing unmethylated G–G mismatches with their C–G counterparts are shown in (H, pH 5.4, 25 mM NaCl and 25°C). Errors in Δ*G_syn-anti_* were obtained by propagating the errors for the individual samples from triplicate measurements (see ‘Materials and Methods’).

Our results suggest that A(*syn*)-U and G(*syn*)-C^+^ HG bps are energetically disfavored in RNA owing to A-form dependent steric clashes of the *syn* base. However, based on X-ray crystallography and solution-state NMR, purine-purine mismatches form *syn-anti* HG bps in both B-DNA and A-RNA duplexes (33–38). Indeed, based on an analysis of crystal structures in the PDB, we identified 69 *syn-anti* purine-purine mismatches in duplex DNA and RNA out of a total of ∼10,000 purine-purine mismatches (Supplementary Table S3) that are listed in Supplementary Table S4. Examination of the torsion angles shows that in A-RNA, steric clashes between the *syn* purine base and backbone in these mismatches are alleviated by changing backbone torsion angles α and γ from canonical *gauche t*o non-canonical *trans* values while retaining a C3′-endo sugar pucker (Figure 5A). Such re-arrangements were not observed in B-DNA (Supplementary Figure S5A). Furthermore, all other backbone torsion angles remained similar to those of canonical WC bps in DNA and RNA helices (Supplementary Figure S5). Prior NMR (84) and computational (85) studies have shown that isolated NTPs energetically favor *gauche* α and γ torsions as compared to *trans*. Thus, the backbone conformational changes toward *trans* needed to accommodate *syn* purines could represent an additional energetic cost (ΔΔG*_syn-anti_*) for forming HG bps in A-RNA compared to B-DNA. If this were true, replacing a WC G–C bp with a HG G–G mismatch should be more destabilizing for A-RNA as compared to B-DNA. We chose G–G mismatches as a model system owing to their tendency to adopt well defined *syn-anti* or *anti-syn* base pairing geometries (34–37), as opposed to A–G which can adopt alternative conformations such as *anti-anti* (38,86) or A–A which are not stably hydrogen bonded (87,88). While there have been studies examining the effects of G–G mismatches on duplex stability (89,90), none have systematically compared the effects for the same sequence contexts in DNA and RNA. Thus, we tested this prediction using G-G mismatches placed in two different sequence contexts A_2_ and gc, that have been previously characterized by NMR (15) (Figure 5B).

NMR spectra show that the G-G mismatches form HG bps (Figure 5C) in both gcDNA and gcRNA based on the observation of two upfield shifted imino protons characteristic of HG bps (Figure 5D) (36,37). Because the sequence around the G–G mismatch is symmetric, the NMR signals arise from both G4(*syn*)–G13(*anti*) as well as G4(*anti*)–G13(*syn*) conformations (Figure 5C) that are degenerate with respect to chemical shifts (G4(*syn*) = G13(*syn*) and G13(*anti*) = G4(*anti*)) and are in slow to intermediate exchange on the NMR chemical shift timescale as evidenced by line broadening in the aromatic and NOESY spectra (Supplementary Figures S6A and B).

To simplify spectra and aid the thermodynamic analysis, we introduced m^1^G4 so as to specifically stabilize the m^1^G4(*syn*)–G13(*anti*) HG bp in gcRNA and gcDNA, as verified based on the m^1^G-C8 chemical shifts (Supplementary Figures S6C and S6D) (70). As expected, in both gcRNA and gcDNA, methylation of G4 resulted in disappearance of the G(*syn*)-H1 imino resonance owing to its replacement with a methyl group (Figure 5D), while minimally perturbing the other imino resonances. Taken together, these results show that it is possible to accommodate a *syn* guanine base in A-RNA when it is base-paired with another guanine.

Based on optical melting experiments, m^1^G(*syn*)-G HG bps destabilized A-RNA duplexes relative to their WC C–G counterparts by 5.8–7 kcal/mol, which can be compared to 3.0–4.3 kcal/mol in the case of DNA duplexes (Figure 5E). The greater destabilization of RNA compared to DNA is robustly observed in the presence of magnesium (Figure 5F), under low salt conditions (Figure 5G), for unmodified G–G mismatches (Figure 5H), in the presence of potassium (Supplementary Table S2) and for alternative sequence contexts (Supplementary Figure S6E). Consistent with the adoption of an energetically unfavorable backbone conformation, the increased destabilization in the case of RNA is seen to be enthalpically driven (Supplementary Table S2). Furthermore, the energetic cost of the α–γ transition as obtained from calculations on model compounds 1.6–7 kcal/mol (85) is in reasonable agreement with the differences in energetic stabilities of m^1^G(*syn*)-G(*anti*) mismatches relative to WC C–G bps (ΔΔ*G_syn–anti_ =* (5.8–7)-(3–4.3) = 1.5–4 kcal/mol*)* in A-RNA versus B-DNA measured using optical melting experiments (Figure 5E–H, Supplementary Table S2). These results are also consistent with the observation that purine nucleotides with C3′-endo sugars have a reduced tendency to adopt a *syn* conformation of the base (30–32). Taken together, our results indicate that accommodation of the *syn* purine accounts for 1.5–4 kcal/mol destabilization of purine-pyrimidine and purine-purine HG bps in A-RNA compared to B-DNA.

### Movement of bases to form HG h-bonds is energetically more costly in A-RNA compared to B-DNA

The above results show that the steric clashes involving *syn* purines in A-RNA can be resolved through changes in the backbone α and γ torsion angles in the context of purine-purine mismatches. In principle, similar conformational adjustments could be used to accommodate m^1^rA(*syn*)-U and m^1^rG(*syn*)-C^+^ HG bps in A-RNA. However, unlike G-G mismatches, m^1^rA-U and m^1^rG-C do not form HG bps in A-RNA, rather they form partially melted conformations generally with an *anti-anti* geometry (15). This suggests that there may be additional energetic costs in A-RNA for forming purine-pyrimidine versus purine-purine HG bps. One important distinction between the purine-pyrimidine and purine-purine HG bps is that in the former, the two bases have to come into closer proximity by ∼2 Å to enable the formation of h-bonds (Figure 1B) (16,56). In contrast, for the larger purine-purine mismatches, such a translation of bases is not required (Supplementary Figure S5A). We recently visualized the conformational changes in DNA that drive movement of the bases to form A(*syn*)-T HG bps using NMR (55,71). Interestingly, similar conformational changes were also observed in MD simulations of m^1^A(*syn*)-T HG bps in B-DNA duplexes (55). Since it is not feasible to experimentally measure the energetic cost of moving the bases, we used MD simulations to test whether the translation of the *syn* adenine and its base pairing partner uridine to form HG h-bonds is energetically more costly in A-RNA as compared to B-DNA, and could contribute to HG bp instability in RNA.

**Figure 6.**
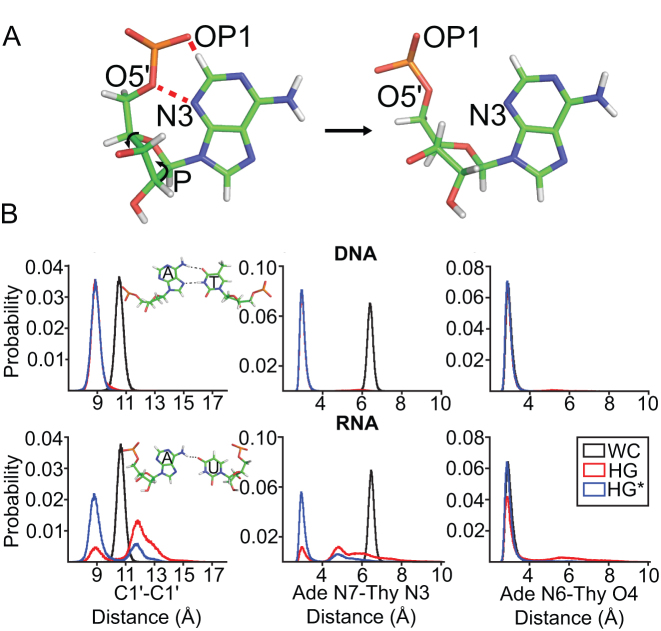
Translation of the bases to form HG h-bonds is energetically more costly in A-RNA as compared to B-DNA. (**A**) Steric clashes (red dashes) between the *syn* purine base and backbone in A(*syn*)-U HG bps are accommodated by changing the sugar pucker away from C3′-endo to C4′-exo, in the MD simulations. (**B**) Histograms of the C1′-C1′ and h-bond distances for WC and A16(*syn*)-T/U9 bps in A_6_-DNA (bsc0) and A_6_-RNA obtained from MD simulations. HG and HG* refer to independent simulations of A_6_-DNA and A_6_-RNA with different starting geometries in which the A16(*syn*)-T/U9 bp forms a HG bp and is in a HG* conformation (in which the *syn* adenine and the complementary T/U are not in base pairing proximity) respectively. Shown in inset to the C1′–C1′ distance panel are representative structural snapshots of the A16(*syn*)-T/U9 bp obtained from the simulations.

Simulations were initiated from B-DNA and A-RNA duplexes (A_6_ sequence context) containing a single A16(*syn*)-T/U9 HG bp using the AMBER simulation package (see ‘Materials and Methods’). We analyzed the C1′-C1′ distance and the stability of the HG h-bonds throughout the course of the simulation. In particular, a C1′–C1′ distance <9.5 Å, χ angle for the *syn* adenine between 0° and 90°, and h-bond donor–acceptor distances < 3.5 Å (56) were used to define the formation of a HG bp. Similar results were also obtained when an angle cutoff (hydrogen–donor–acceptor angle < 30°) was additionally used to define the formation of a hydrogen bond (Supplementary Table S6). In the case of DNA, the HG bp remained stably formed (Figure 6B, Supplementary Tables S6 and S7, Supplementary Figure S8) for >90% of the simulation time in all three force fields tested (bsc0, bsc1 and OL15). Transitions to WC bps were not observed during the simulations, which were short (∼1 μs) compared to the lifetime (0.1–1 ms) of the HG bp in duplex DNA (17,18).

Interestingly, the A–U bp in the RNA simulations did adopt hydrogen bonded HG conformations, in which the steric clashes with the *syn* purine base were resolved through changes in sugar pucker (Figure 6A), unlike the changes in the α and γ torsions observed in crystal structures of HG purine-purine mismatches (Figure 5A). Although we cannot rule out that this could be caused due to the tendency of the AMBER force fields to destabilize *trans* α-γ conformations (91), this suggests that there could be multiple ways of sterically accommodating the *syn* purine base in RNA.

Movement of the bases to form HG h-bonds (Supplementary Figure S9A) in A_6_-RNA was accompanied by localized changes in sugar pucker (away from C3′-endo to C4′-exo), β torsion angle (towards 180°) of the *syn* purine nucleotide, α torsion angle of its 3′ neighbor (Supplementary Figure S9B), and over-twisting of the helix about the HG bp (by ∼6° relative to the WC bp, Supplementary Figure S9C). These characteristics of the HG bp in A-RNA obtained from the MD simulations are subtly different from the changes accompanying HG bp formation in B-DNA (55,71), which involve changes in the sugar pucker, ϵ and ζ torsion angles of the *syn* purine and its 5′ neighbor, along with under-twisting and major groove directed kinking of the helix at the HG bp.

However in A_6_-RNA, only ∼40% of conformations sampled during the course of the simulation have bases with constricted C1′-C1′ distances that are positioned for HG hydrogen bonding (Supplementary Table S6). For the remainder of the time, the bases splayed apart to adopt conformations in which the C1′-C1′ distance is no longer constricted, and the adenine N7-uracil N3 (N7–H3–N3) h-bond is broken (Figure 6B inset, Supplementary Tables S6 and S7). These conformations no longer feature changes in the α and β torsions, and sugar pucker, that are required to move the bases/constrict the backbone for HG pairing in RNA (Supplementary Figure S9B). Based on the simulations, the energetic cost to move the bases to form A(*syn*)-T/U HG type h-bonds is estimated to be higher for A-RNA as compared to B-DNA by ΔΔ*G*_constrict_ = 0.3 – (−3.1) = 3.4 kcal/mol (Supplementary Table S6). In contrast, the stability of the HG h-bonds was found to be similar for G–G mismatches in B-DNA and A-RNA (Supplementary Figure S9D), suggesting that the instability of A(*syn*)-U HG bps in the simulations of RNA is not an artifact purely caused due to the presence of a *syn* base. Qualitatively similar results were also obtained with simulations of A_6_-DNA and A_6_-RNA containing G(*syn*)-C^+^ HG bps and A_2_-DNA and A_2_-RNA containing A(*syn*)-T HG bps respectively; the constriction of the backbone was seen to be energetically more costly in RNA as compared to DNA by ΔΔ*G*_constrict_ = 2.1 – (−1.9) = 4 kcal/mol and ΔΔ*G*_constrict_ = 0.2 – (−2.8) = 3 kcal/mol (Supplementary Table S6).

If constriction of the backbone to form HG h-bonds is energetically more costly in A-RNA as compared to B-DNA, one might expect that U-U wobble mismatches, which also require closer proximity of bases relative to WC bps, would also be more destabilizing in A-RNA as compared to T-T mismatches in B-DNA, assuming that the absence of the methyl group on Uracil in RNA does not contribute to the energetics of the process. Indeed, based on an analysis of nearest neighbor thermodynamic parameters (89,90), replacing a G–C WC bp with a U–U wobble results in a greater degree of destabilization of A-RNA (5.6–7.1 kcal/mol) as compared to replacing the G–C WC bp with a T–T wobble mismatch in B-DNA (3.8–5.2 kcal/mol) (Supplementary Figure S9E). The difference in free energy (1.8 ± 0.6 kcal/mol) is in reasonable agreement with the added energetic cost of constricting the backbone to form HG h-bonds in A-RNA compared to B-DNA calculated using MD (∼3.4–4.0 kcal/mol).

## DISCUSSION

Our results indicate that the A-form geometry and not the 2′-hydroxyl at the purine nucleotide, is primarily responsible for the greater instability of HG bps relative to WC bps in A-RNA as compared to B-DNA. Although direct influences on HG bp stability due to a single hydroxyl group in RNA are small, the combined presence of multiple hydroxyl groups on both strands indirectly suppresses HG bps by enforcing the A-form helical geometry. In sharp contrast, by loosening the helical geometry of the A-form, we successfully induced a stable m^1^G(*syn*)-C^+^ HG bp in RNA (Figure 4). To our knowledge, this represents the first observation of a purine-pyrimidine HG bp in helical RNA under solution conditions.

**Figure 7.**
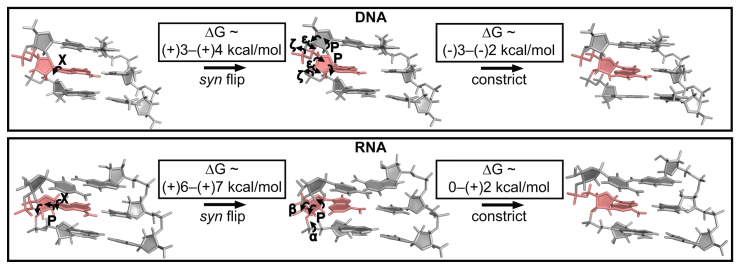
Mechanism of HG bp accommodation in DNA and RNA. The energetic cost for forming HG bps can be decomposed into contributions from flipping the base from an *anti* (WC) to a *syn* conformation followed by movements of the bases to form HG type h-bonds. Both steps are energetically more costly in RNA as compared to DNA. Note that the *syn* base in RNA could also be accommodated by changing the α and γ torsions to *trans*. The geometric changes accompanying HG bp formation in DNA are based on NMR studies (55,71), while those in A-RNA are based on MD simulations in this study.

Our results also suggest that there are two contributions to the energetic penalty (ΔΔ*G*_HG-WC_) for forming HG bps in A-RNA as compared to B-DNA. The first is the added energy cost of ∼1.5–4 kcal/mol (ΔΔG*_syn–anti_*) associated with conformational changes in the α and γ torsion angles (Figure 5A) or sugar pucker (Figure 6A) needed to resolve steric clashes with the *syn* purine base (Figure 7). We used differences in the relative stabilities of C-G WC and m^1^G(*syn*)-G HG bps between RNA and DNA to estimate this energetic term. This assumes that the *syn* purine base adopts similar conformations in purine-purine and purine-pyrimidine HG bps (prior to backbone constriction) in both B-DNA and A-RNA. A comparison of χ angles of *syn* purines in purine-purine HG mismatches, and in G(*syn*)-C^+^ as well as A(*syn*)-T HG bps in DNA shows that this is indeed the case (Supplementary Figure S5A). Furthermore, it is also assumed that the effects of replacing the C that is paired with the G (in C–G WC bps) with a G (in G–G HG bps) are similar between RNA and DNA. Interestingly, an analysis of purine-purine mismatches in the PDB (see ‘Materials and Methods’) suggests that this replacement might be accompanied by a loss of stacking interactions in the case of RNA compared to DNA (Supplementary Figure S6F) and may also contribute to their instability as compared to DNA. Additional studies are needed to dissect how differences in stacking interactions contribute to the energetic cost of accommodating a *syn* base. These results generalize the finding that HG bps are energetically disfavored in A-RNA compared to B-DNA by including purine-purine HG mismatches even though the differences in energetic cost are lower compared to purine-pyrimidine HG bps.

Based on MD simulations, a second contribution is an added energetic penalty of 3–4 kcal/mol (ΔΔG_constrict_) applicable only for purine-pyrimidine HG bps (in RNA as compared to DNA) that is associated with translation of the bases to form HG h-bonds (Figure 7). Purine-purine HG bps which do not require movements of the bases (Supplementary Figure S5A) would not need to pay this energetic cost. Coupled with the potentially higher energy of alternative conformations such as *anti–anti*, this rationalizes why G-G mismatches form HG bps in A-RNA whereas m^1^A-U and m^1^G-C adopt unstably paired conformations with lack of base pairing around the site of incorporation (15). Although the instability of the HG bp was evident in the simulations of RNA, they were unable to capture the experimentally observed instability of WC bps neighboring the site of *N*^1^-methylation (15), potentially due to the occurrence of opening events on time scales longer than those employed in the simulations (Supplementary Figure S10).

Interestingly, with the exception of a couple of recent NMR studies (36,37), prior NMR studies reported that that G-G mismatches adopt alternative *anti-anti* and *trans* hydrogen bonded *syn-anti* conformations (Supplementary Figure S6G) (92–95). The alternative bp geometries proposed in the prior studies likely arose from misinterpretation of weak intra-nucleotide H1′-H8 cross peak intensities as being indicative of an *anti* conformation for the base (93,94) without accounting for broadening due to conformational exchange between *syn-ant*i and *anti-syn* HG conformations. Furthermore, the two upfield shifted imino proton signals (Figure 5D) were misinterpreted as arising from a *trans* hydrogen bonded bp conformation (Supplementary Figure S6G) in which both the imino protons are hydrogen bonded to the O6 base atoms (92,95), without considering the possibility that the imino peak from a *syn* base that is exposed to solvent can also be observed as reported for G–C HG bps (96–98). In contrast, more recent studies that concluded a HG bp geometry consistent with crystallographic studies of G–G mismatches (34,35) (Figure 5C), employed sequence contexts that are less prone to line broadening due to conformational exchange (36) or utilized 8-bromo guanine substitutions to stabilize the *syn* conformation of the guanine base (37). By using carbon chemical shifts and m^1^G substitutions to minimize conformational exchange and line broadening, we have also circumvented the above-mentioned complications, and obtained definitive evidence for G(*syn*)–G(*anti*) HG bps in dynamic equilibrium.

Based on the total energetic cost for accommodating the *syn* purine and translation of the bases to form h-bonds, we estimate that A(*syn*)-U and G(*syn*)-C^+^ HG bps are disfavored in A-RNA compared to B-DNA by a combined amount (ΔΔ*G*_HG-WC_) of 4.5–8.0 kcal/mol. This exceeds the value of ΔΔ*G*_N1methyl-WC_ (1.1–4.7 kcal/mol) obtained from optical melting experiments on *N*^1^-methylated purines in A-RNA and B-DNA (15). This discrepancy can be rationalized by the observation that the *N*^1^-methylated purines in RNA do not form HG bps, but rather adopt partially melted *anti-anti* conformations at least in the case of m^1^A (15). Thus, melting measurements on these constructs likely only provide a lower limit for the destabilization of the HG state in A-RNA compared to B-DNA. Therefore, instead of paying the energetic costs of flipping the base to *syn* and constricting the backbone, *N*^1^-methylated purines in RNA prefer to adopt alternative *anti*-anti conformations (15) possibly stabilized by a single h-bond and more optimal stacking interactions. Additional experiments are needed to examine the robustness of the magnitudes of the obtained energies under more extensive experimental conditions such as higher pH and salt concentrations.

The results from this study have important biological implications. Firstly, our results provide key insights into the origins of the destabilizing effects of *N*^1^-methylated purines in RNA, which constitute an important form of post-transcriptional regulation (21). We find that m^1^A and m^1^G destabilize RNA duplexes in a context dependent manner. They are most destabilizing for WC bps within the interior of A-form helices wherein base pairing is disrupted completely (15). The destabilizing effects are weaker for WC bps near junctions (Figure 4E), and other mismatches (Figure 5G–H, Supplementary Table S2), where they can be accommodated via non-canonical base pairing modes. The low propensity for A-RNA to accommodate *syn* purine bases has also been proposed to play key roles in governing the propensity of RNA to form G-quadruplexes with parallel topologies that exclusively contain *anti* bases in the G4 tetrad region, as opposed to those that are anti-parallel which contain a mixture of *syn* and *anti* guanines (99).

Secondly, they provide new insights into how dNMP and rNMP substitutions affect the structures of A-RNA and B-DNA. Such substitutions occur in nature as DNA and RNA polymerases misincorporate rNTPs and dNTPs respectively (13,14). Prior NMR studies indicated that isolated rNMPs in DNA duplexes adopt a C3′-endo sugar pucker (73,74). In contrast, we find that in A_6_-DNA which features an A-tract, they retain a C2′-endo sugar pucker (Figure 3C–D). These results suggest that accommodation of rNMPs in B-DNA could occur by two independent mechanisms - changing the sugar pucker of the rNMP toward C3′-endo or changing the backbone/phosphate conformation while retaining the C2′-endo sugar pucker, with the preference for either mode being determined by the sequence context. Further studies are required to assess such sequence-dependent mechanisms. Our NMR analysis also indicates that purine dNMPs in A_6_-RNA retain an A-form geometry. These results are consistent with prior CD (100) and IR (101) studies showing minor effects of isolated dNMP substitutions on the conformation of RNA helices and NMR studies (102) showing that a minimum of 4 dNMPs are needed to nucleate a C2′-endo conformation in an RNA–DNA hybrid. This suggests that in contrast to the behavior of rNMPs in B-DNA, the impact of dNMPs in A-RNA is likely to be less sequence dependent.

Lastly, the findings in this study may also have implications for understanding the sequence dependence of errors generated during transcription. In particular, it has been proposed that purine-purine mismatches can give rise to transversion mutations (A–T→C–G or G–C→T–A) via the adoption of tautomeric HG conformations that mimic the shape of WC bps (103,104). Our results suggest that RNA-DNA hybrid sequences that have an increased propensity to adopt conformations biased towards the A-form, such as those with purine rich RNA strands (105–107), would potentially be less capable of harboring these mismatch conformations and consequently would have less transversion errors, relative to those hybrids that have a reduced tendency to be A-form like, such as those with pyrimidine rich RNA strands. Furthermore, by virtue of being more destabilizing, purine-purine mismatches in sequence contexts biased to the A-form would also be likely to be proofread (108) more effectively. Additional experiments are needed to test these hypotheses.

## DATA AVAILABILITY

In-house Python scripts for generating the datasets of WC bps and purine-purine mismatches used in this study can be found at https://github.com/alhashimilab/CanonicalBP and https://github.com/alhashimilab/MismatchPDB respectively.

## Supplementary Material

Supplementary Data
