# Supplementary material for: Why are Hoogsteen base pairs energetically disfavored in A-RNA compared to B-DNA?
Source: Nucleic Acids Res. 2018 Oct 4;46(20):11099–114. doi: 10.1093/nar/gky885 (PMC6237737; doi:10.1093/nar/gky885)
Supplement: Supplementary Data [file gky885_supplemental_files.pdf]

Supplementary Information

Why are Hoogsteen base pairs energetically disfavored in A-RNA compared to B-DNA?

Atul Rangadurai[1], Huiqing Zhou[1,4], Dawn K. Merriman[2], Nathalie Meiser[3], Bei Liu[1], Honglue Shi[2], Eric S. Szymanski[1] and Hashim M. Al-Hashimi*[1,2]

1. Department of Biochemistry, Duke University School of Medicine, Durham, NC, USA

2. Department of Chemistry, Duke University, Durham, NC, USA

3. Goethe University, Institute for Organic Chemistry and Chemical Biology, Frankfurt am Main, Germany

4. Present address - Institute for Biophysical Dynamics, University of Chicago, Chicago, IL, USA

**To whom correspondence should be addressed: hashim.al.hashimi@duke.edu*

Tel: 919-660-1113

Supplementary Figures

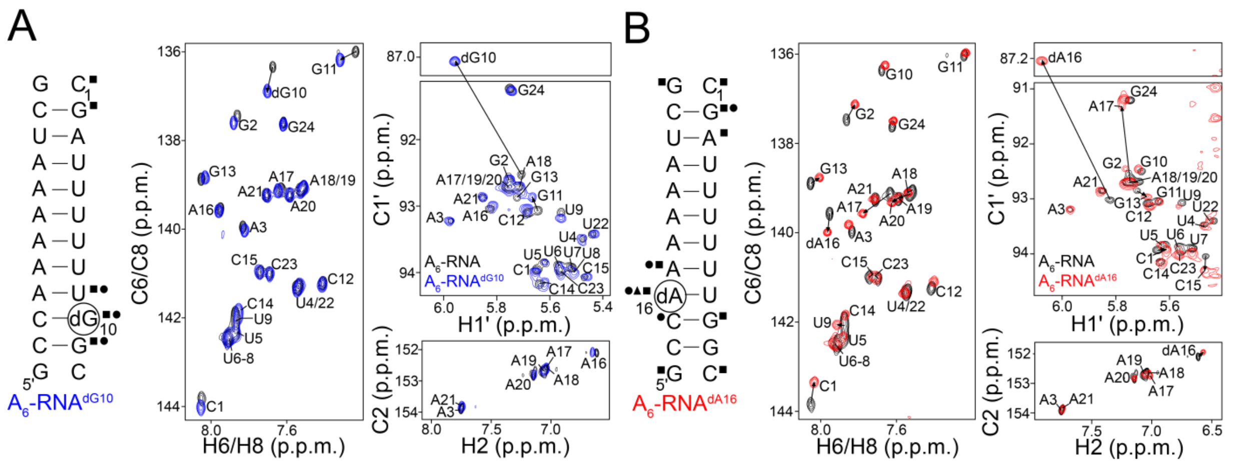

Figure S1. NMR chemical shift perturbations induced by purine dNMP incorporations in A_6-RNA. Comparison of 2D CH HSQC spectra of aromatic (C6/C8/C2-H6/H8/H2) and sugar (C1′-H1′) resonances of (A) A_6-RNA^{dG10} (blue) and (B) A_6-RNA^{dA16} (red) with unmodified A_6-RNA (black) at pH 5.4, 25 mM NaCl and 25 °C. Significant chemical shift perturbations (see 'Materials and Methods') on removal of the 2′-hydroxyl, for the C8-H8/C6-H6, C2-H2 and C1′-H1′ pairs of resonances are denoted using black squares, triangles and circles respectively, on the duplexes. The dNMP residues are also indicated using black circles.

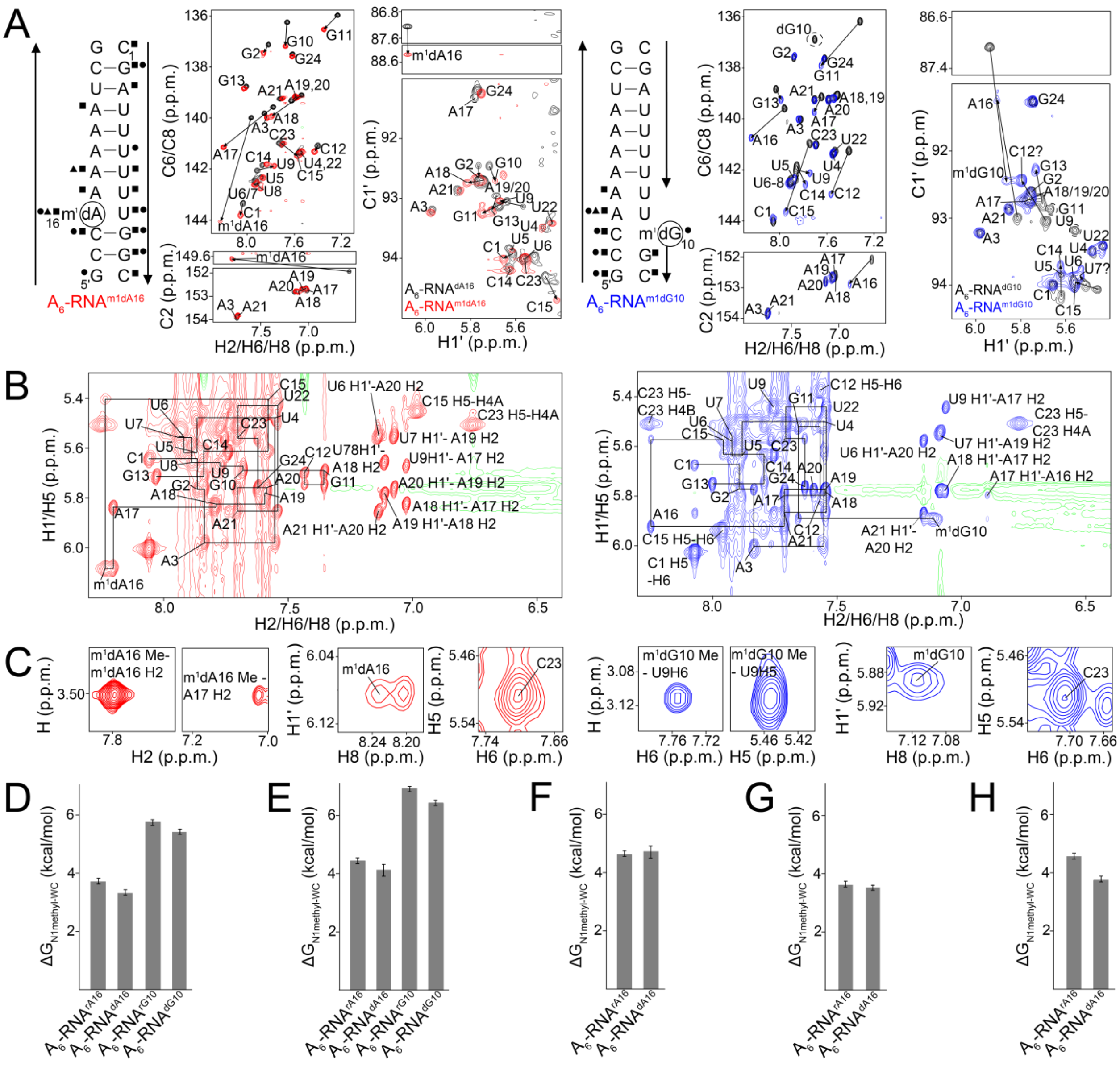

Figure S2. Removal of the 2′-hydroxyl from a purine residue does not rescue HG bp formation in A_6-RNA. (A) Comparison of 2D CH HSQC spectra of aromatic (C6/C8/C2-H6/H8/H2) and sugar (C1′-H1′) resonances of A_6-RNA^{m1dA16} (red) and A_6-RNA^{m1dG10} (blue) with their unmethylated counterparts, A_6-RNA^{dA16} and A_6-RNA^{dG10} (black). Significant chemical shift perturbations (see 'Materials and Methods') on N^1-methylation for the C8-H8/C6-H6, C2-H2 and C1′-H1′ pairs of resonances are denoted black using squares, triangles and circles respectively, on the duplexes. The dNMP residues are indicated using black circles. NOE connectivities are

indicated using black arrows. Resonances that are broadened out of detection are indicated using dotted circles on the spectra. The downfield shifted m^{1}dA16-C8 in A$_6$-RNA^{m1dA16} is likely due to protonation of the base on *N*^{1}-methylation and not due to the adoption of a *syn* conformation (1,2). The ~5.5 ppm downfield shift of dG10-C1′ on *N*^{1}-methylation is consistent with a *syn* conformation for the methylated base (3). (B) The H1′-H8 region of the 2D NOESY spectra of A$_6$-RNA^{m1dA16} (red) and A$_6$-RNA^{m1dG10} (blue). The H1′-H8 base-backbone NOE walk is indicated using black lines. For A$_6$-RNA^{m1dA16}, C15 H1′-m^{1}dA16 H8 NOE connectivity is observed, indicative of an *anti* conformation for the m^{1}dA16 base. (C) NOE connectivities between the *N*^{1}-methyl group in A$_6$-RNA^{m1dA16} (red) and A$_6$-RNA^{m1dG10} (blue) and the neighboring base protons (A17-H2 for A$_6$-RNA^{m1dA16} and U9-H5/H6 for A$_6$-RNA^{m1dG10}) are consistent with an *anti* and *syn* conformation for m^{1}dA16 and m^{1}dG10 respectively. Also shown is a comparison between the intra-nucleotide H1′-H8 NOE cross peaks for m^{1}dA16 (red) and m^{1}dG10 (blue) in A$_6$-RNA^{m1dA16} and A$_6$-RNA^{m1dG10}, and a reference cytosine (C23). The weak H1′-H8 NOE cross peak for m^{1}dA16 is consistent with an *anti* conformation for the base. In contrast, the weak H1′-H8 NOE cross peak for m^{1}dG10(*syn*) is likely due to exchange broadening. The m^{1}dG10-rC15 bp likely adopts a singly hydrogen bonded m^{1}dG10(*syn)*-C15(*anti*) conformation, although we cannot establish that doubly hydrogen bonded HG conformations are not formed transiently. (D-H) The energetic cost of introducing an *N*^{1}-methyl group ($\Delta G_{N1methyl\text{-}WC}$) estimated from melting experiments on A$_6$-RNA constructs with and without *N*^{1}-methylated purines at the indicated position, in (D) low salt (pH 5.4, 25 mM NaCl and 25 °C), (E) moderate salt (pH 5.4, 150 mM NaCl and 25 °C), (F) presence of magnesium (pH 5.4, 150 mM NaCl, 3mM $MgCl_2$ and 25 °C), (G) neutral pH (pH 6.8, 25 mM NaCl and 25 °C) and (H) presence of potassium (pH 5.4, 150 mM KCl and 25°C) are similar in the presence and absence of the 2′-hydroxyl, suggesting that its removal does not rescue HG bp formation under these conditions. Errors in $\Delta G_{N1methyl\text{-}WC}$ were obtained by propagating the errors from triplicate measurements (see ‘Materials and Methods’).

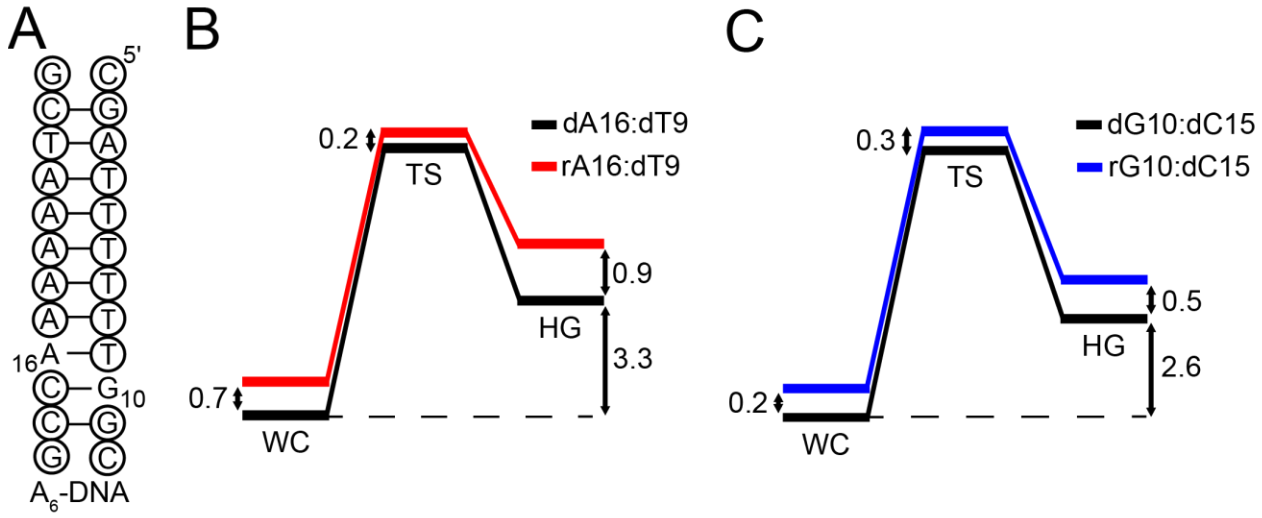

Figure S3. Single rNMP incorporations minimally impact the free energy for the formation of HG bps in A_6-DNA, while destabilizing both WC and HG bps. (A) A_6-DNA duplex with the dNMP residues indicated using circles. The sites of rNMP incorporation are A16 and G10. Free energy diagram for the WC to HG transition for (B) A16-T9 and (C) G10-C15 bps in A_6-DNA with and without a 2′-hydroxyl at pH 5.4, 25 mM NaCl and 10 °C, and pH 5.4, 25 mM NaCl and 25 °C respectively. The difference in free energies between the WC base paired duplexes with and without the rNMP incorporation were obtained using optical melting experiments, while the free energy differences between the WC and HG state, and between the WC and transition state (TS) were deduced from RD measurements performed previously (1).

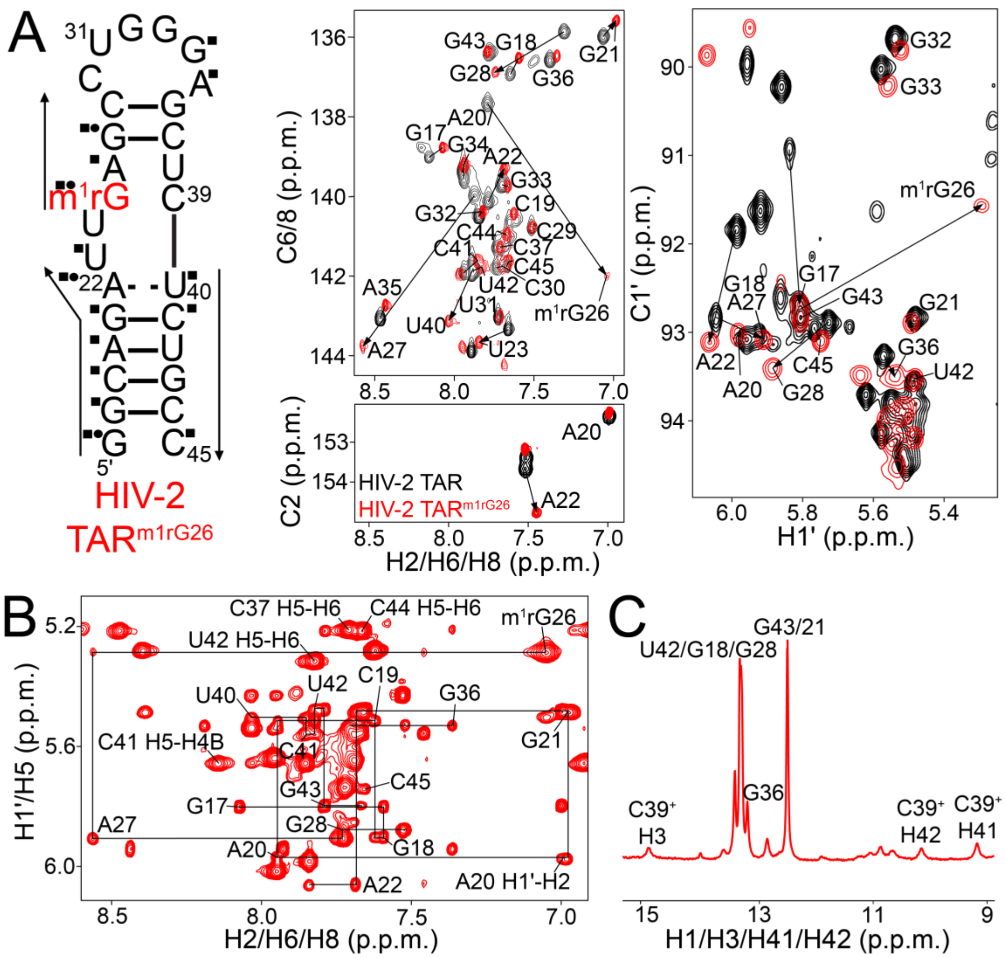

Figure S4. Chemical shift assignments of HIV-2 TAR^{m1rG26}. (A) Comparison of 2D CH HSQC spectra of aromatic (C2/C6/C8-H2/H6/H8) and sugar (C1′-H1′) resonances of (A) HIV-2 TAR^{m1rG26} (in red) with unmodified HIV-2 TAR (black, spectra obtained from Merriman *et al.*,(4)) at pH 5.8, 25 mM NaCl and 25 °C. Significant chemical shift perturbations (see 'Materials and Methods') on N^1-methylation of G26, for the C8-H8/C6-H6, C2-H2 and C1′-H1′ pairs of resonances are denoted using black squares, triangles and circles respectively, on the secondary structure. The H1′-H8 base-backbone NOE walk is indicated using black arrows. (B) The H1′-H8 region of the 2D NOESY spectra of HIV-2 TAR^{m1rG26} (red) at pH 5.8, 25 mM NaCl and 25 °C. (C) ^{1}H 1D spectrum of the imino region of HIV-2 TAR^{m1rG26} (red) at pH 5.8, 25 mM NaCl and 10 °C showing the downfield shifted NH1/NH2 amino protons of protonated C39$^+$.

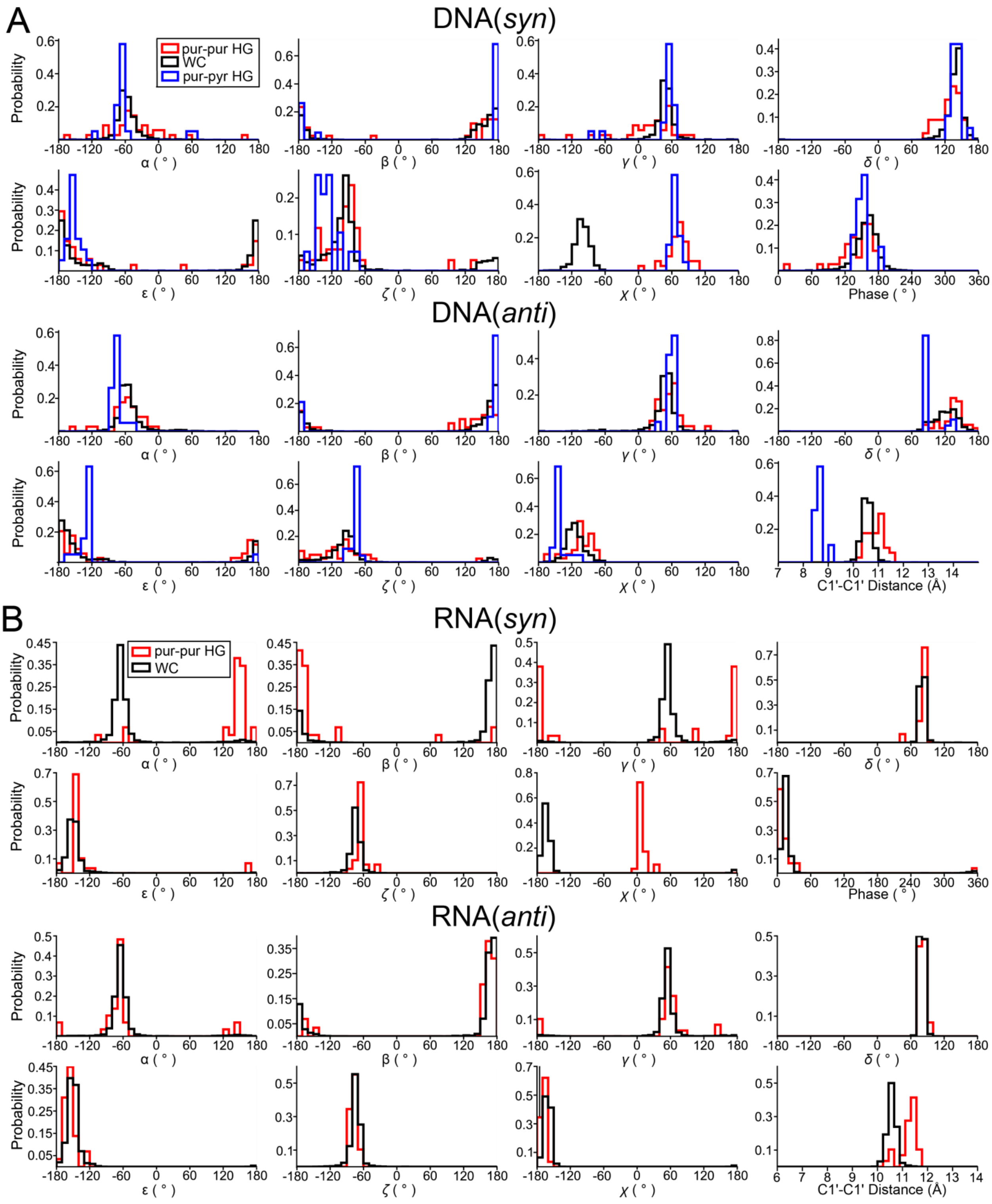

A
DNA(syn)
pur-pur HG
WC
pur-pyr HG
Probability
α (°)
β (°)
γ (°)
δ (°)
ε (°)
ζ (°)
χ (°)
Phase (°)
DNA(anti)
C1'-C1' Distance (Å)
B
RNA(syn)
RNA(anti)

Figure S5. Accommodation of purine-purine HG bps in DNA and RNA helices.

Histograms of endocylic torsion angles and C1′- C1′ distances of purine-purine HG (pur-pur HG) mismatches (red) in (A) DNA and (B) RNA obtained from a survey of crystal structures in the PDB (see 'Materials and Methods'). The torsion angles of the *syn* and *anti* purine in the pur-pur HG mismatch are compared to those of the *anti* purine and *anti* pyrimidine in WC bps (black) respectively, for both DNA and RNA. Also shown for DNA are the endocyclic torsions of purine-pyrimidine HG (pur-pyr HG) bps (blue). The *syn* purine in pur-pur HG mismatches is compared with the *syn* purine in pur-pyr HG bps. The two pur-pur HG mismatches in RNA having a *syn* purine with a *gauche* α-γ conformation in the PDB were reported to have a *trans* α-γ conformation in the associated paper (5).

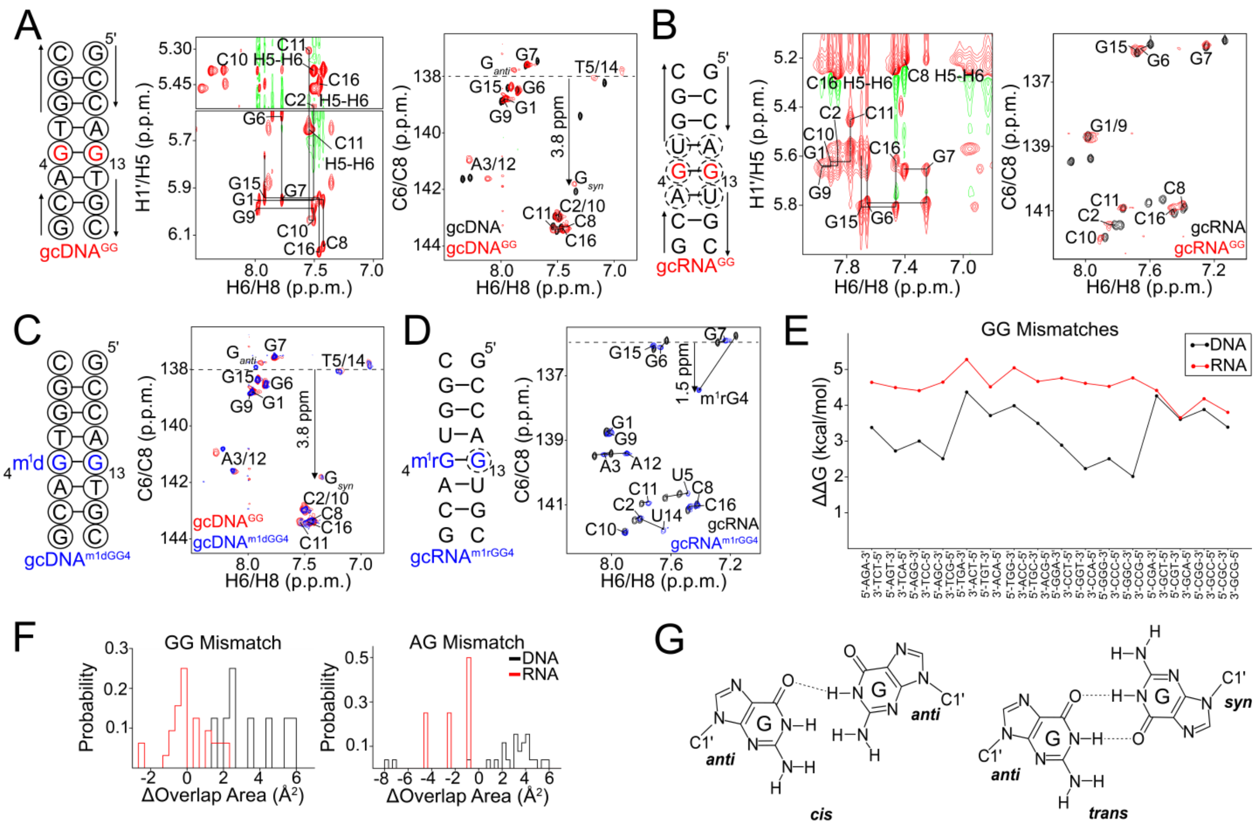

Figure S6. HG G-G/m[1]G-G mismatches in gcDNA and gcRNA. The H1′-H8 region of the 2D NOESY spectra of (A) gcDNA^{GG} (red) and (B) gcRNA^{GG} (red), along with a comparison of their 2D CH aromatic HSQC spectra with gcDNA and gcRNA (black) at pH 5.4, 25 mM NaCl and 10 °C. NOE connectivities are indicated using arrows while residues that are broadened out of detection in the aromatic HSQC spectra are indicated using dotted circles on the duplexes. dNMP residues are indicated using black circles. Comparison of the 2D aromatic (C6/C8-H6/H8) spectra of (C) gcDNA^{m1dGG4} and (D) gcRNA^{m1rGG4} (blue) with gcDNA^{GG} (red) and gcRNA (black) at pH 5.4, 25 mM NaCl and 10 °C and 15 °C, respectively. (E) Differences in the free energies of melting of triplets of base pairs containing G-G and G-C bps as a function of their sequence context, obtained using MELTING (6) (see 'Materials' and Methods'). (F) Differences in the area of overlap (with neighboring bps) between purine-purine HG bps and WC bps, for DNA (black) and RNA (red), computed using 3DNA (7) (see 'Materials and Methods'). (G) Alternative base pairing geometries for G-G mismatches proposed in the literature.

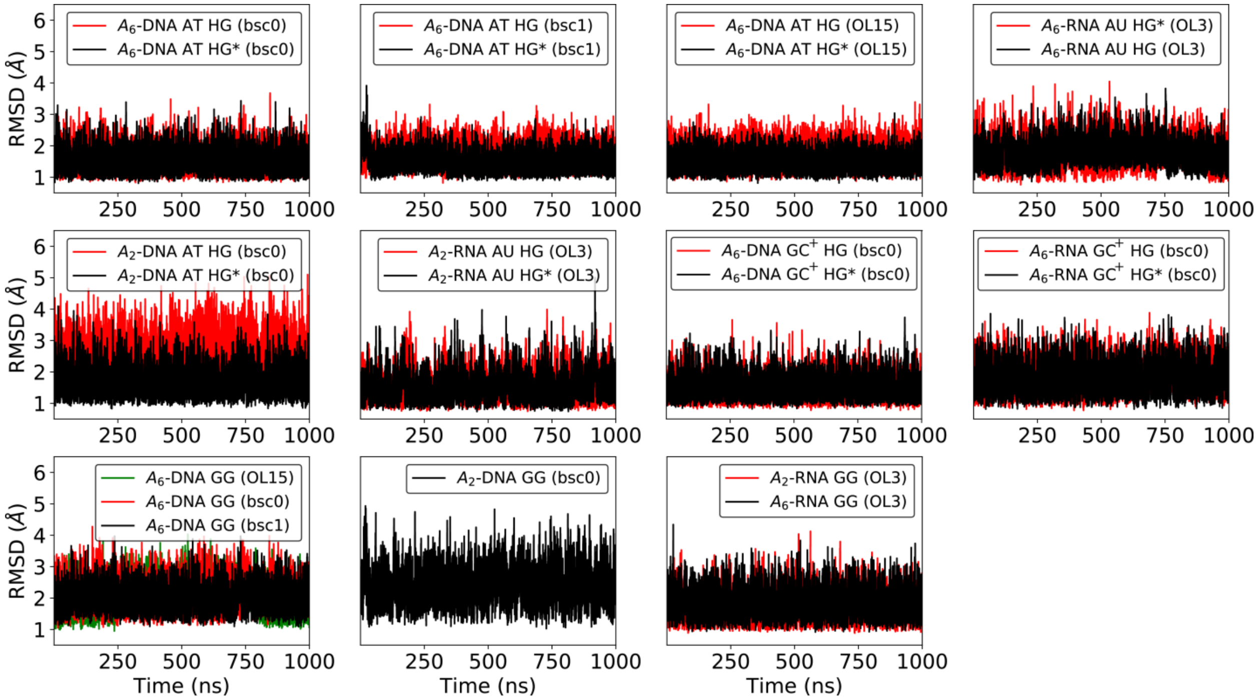

Figure S7. Time evolution of the RMSD during the MD simulations of DNA and RNA. The RMSD is computed for the heavy atoms of the non-terminal residues of the DNA/RNA duplexes.

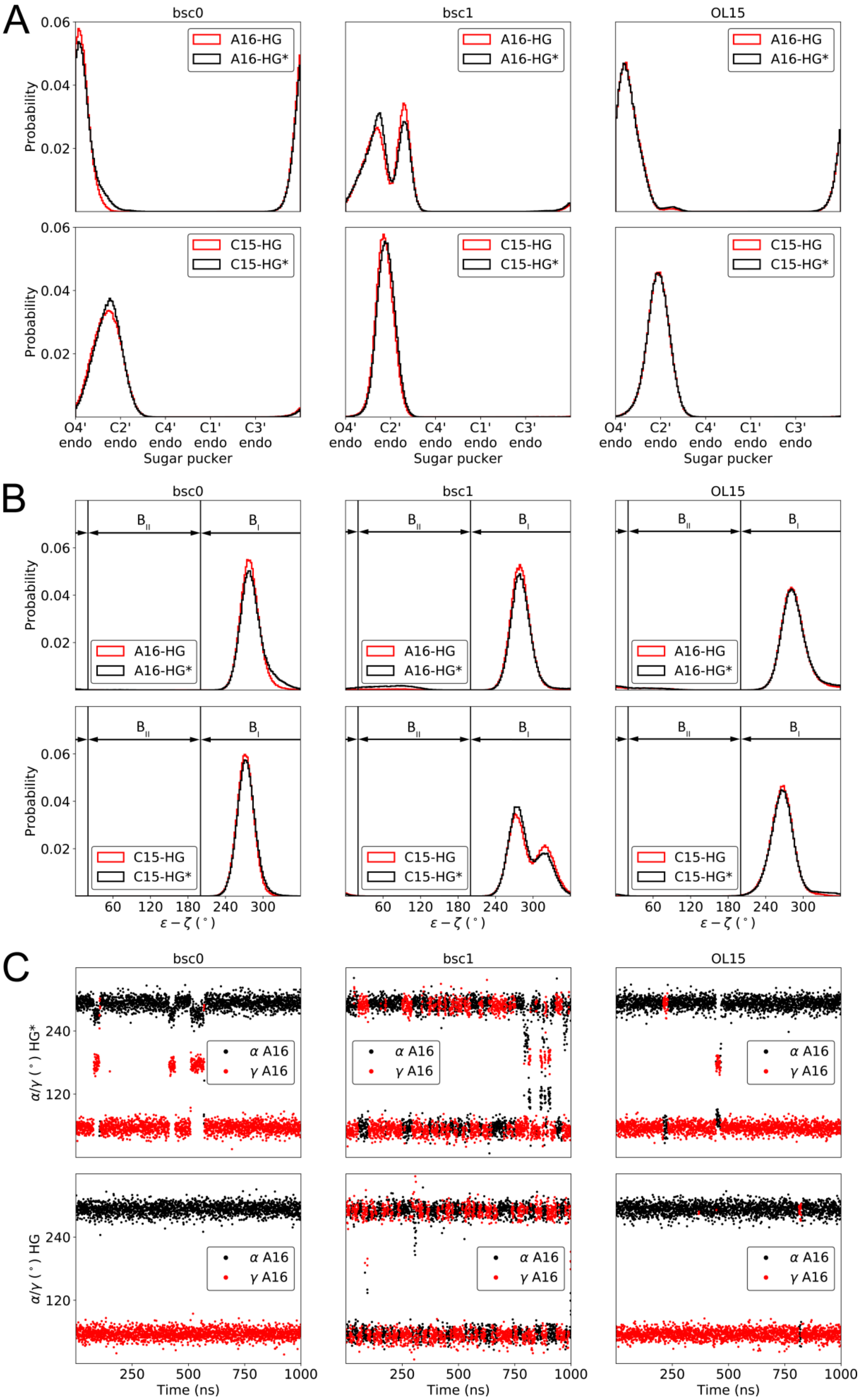

A
bsc0
bsc1
OL15
A16-HG
A16-HG*
C15-HG
C15-HG*
Probability
0.06
0.04
0.02
O4' endo
C2' endo
C4' endo
C1' endo
C3' endo
Sugar pucker
B
B_II
B_I
60
120
180
240
300
ε − ζ (°)
C
α A16
γ A16
α/γ (°) HG*
α/γ (°) HG
240
120
250
500
750
1000
Time (ns)

Figure S8. Accommodation of the A16(*syn*)-T9 HG bp in MD simulations of A_6-DNA with different force fields. (A) 1D histograms of the sugar pucker of A16 and C15 in the MD simulations of A_6-DNA with the bsc0, bsc1 and OL15 force fields. HG and HG* refer to independent simulations of A_6-DNA with a HG and HG* starting conformation of the A16-T9 bp respectively (see 'Materials and Methods' section). A16 predominantly adopts an O4′-endo conformation in simulations with the bsc0 and OL15 force fields in accordance with NMR measurements (8,9), while it adopts a C2′-endo conformation in the bsc1 simulations. C15 predominantly adopts a C2′-endo sugar pucker in simulations with all three force fields, in line with the NMR data (8,9). (B) 1D histograms of the ε-ζ metric, used for classifying phosphate conformations into B_I (ε-ζ < 20° and ε-ζ > 200°) or B_{II} (20° < ε-ζ < 200°), for A16 and C15 in the MD simulations. In accordance with NMR data (8,9), the phosphates of A16 and C15 adopt a predominantly B_I conformation in simulations with all 3 force fields. (C) Variation of the α and γ torsion angles of the *syn* A16 residue during the MD simulations. The bimodal nature of the sugar pucker distribution of A16 in the bsc1 simulations is coupled to the occurrence of frequent α-γ transitions. Frequent α-γ transitions are not seen for the bsc0 and OL15 simulations.

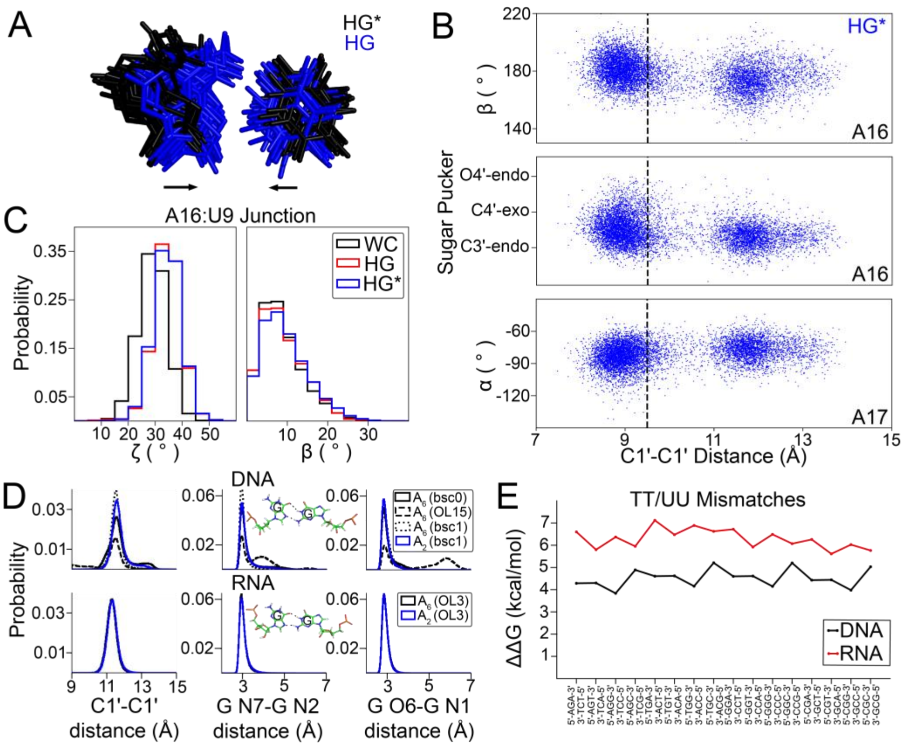

Figure S9. Accommodation of HG bps in MD simulations of A_6-RNA. (A) Superposition (using the heavy atoms of the two neighboring bps) of 20 randomly selected structures of the A16-U9 bp in HG (blue) and HG* (black) geometries. (B) Scatter plots of the C1′-C1′ distance across the A16(*syn*)-U9 bp and the sugar pucker and β torsion angle of A16, and α torsion angle of A17. The dashed line denotes the C1′-C1′ distance cutoff (9.5 Å) used for defining the formation of a HG bp. (C) Histograms of the inter-helical Euler angles (ζ=twist angle, β=bend angle) at the A16-U9 bp, for WC (black) and HG (red/blue) bps, computed as described previously (10). WC, HG and HG* (also in panel B) denote the starting geometry of the A16-U9 bp in the MD simulations. (D) Histograms of the C1′- C1′ and h-bond distances for G-G mismatches in A_6-DNA/A_6-RNA (black) and A_2-DNA/A_2-RNA (blue). The G-G mismatch in the A_6-DNA simulation with the OL15 force field is unstable and adopts a non-hydrogen bonded conformation in which the guanines are stacked on top of each other. (E) Differences in the free energies of melting of triplets of base pairs containing T-T/U-U and G-C bps as a function of their sequence context, obtained using MELTING (6) (see 'Materials' and Methods').

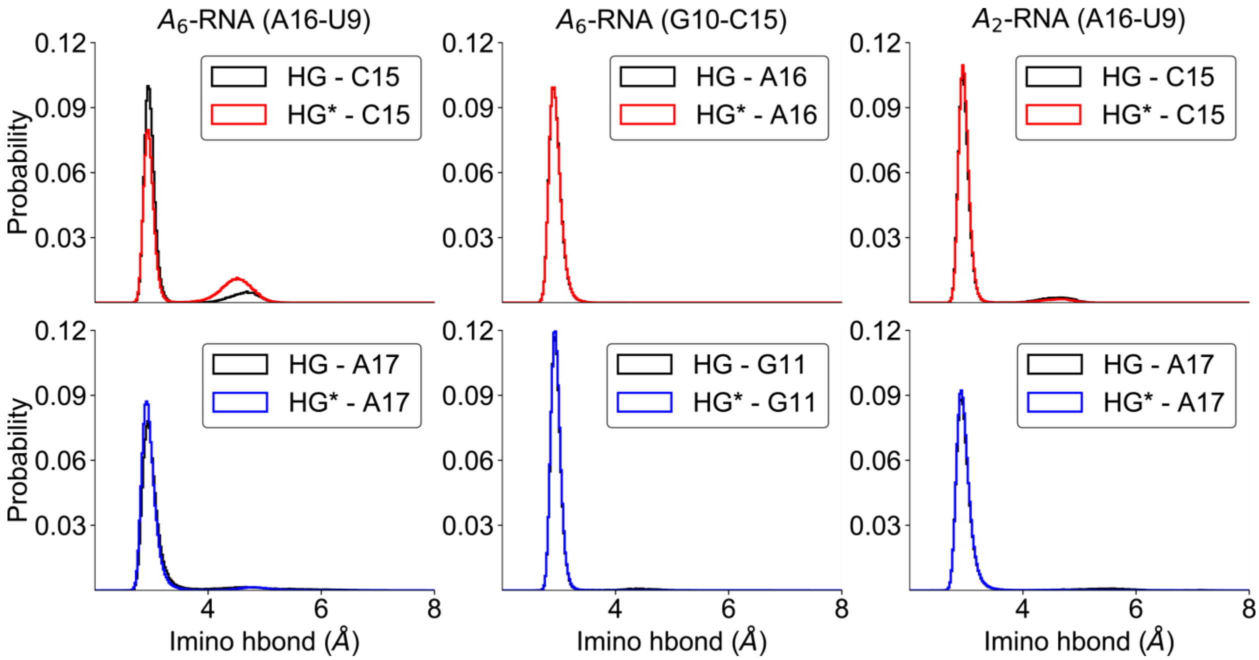

Figure S10. Hydrogen bonding interactions of base pairs neighboring the HG bp in MD simulations of RNA. 1D histograms of the imino hydrogen bond distances (A(N1)-U(N3) or G (N1)-C^+(N3)) for base pairs neighboring the A16(*syn*)-U9 HG bp in A_6-RNA (left), the G10(*syn*)-$C15^+$ bp in A_6-RNA (middle) and the A16(*syn*)-U9 HG bp in A_2-RNA (right), obtained from MD simulations.

Supplementary Tables

Construct	pH	[NaCl] /[KCl] (mM)	[$MgCl_2$] (mM)	C_t (μm)	T_m (°C)	-ΔH (kcal/mol)	-ΔS (cal/mol/K)	$-\Delta G_{25°C}$ (kcal/mol)
A_6-RNA	5.4	25	0	3	40.0±0.1	94.6±1.2	275.5±3.7	12.5±0.1
A_6-RNA^{dG10}	5.4	25	0	3	36.0±0.1	92.8±2.0	273.6±6.4	11.3±0.1
A_6-RNA^{m1rG10}	5.4	25	0	3	18.9±0.5	55.0±2.5	161.8±8.1	6.8±0.1
A_6-RNA^{m1dG10}	5.4	25	0	3	11.2±1.2	41.7±2.4	119.9±7.8	5.9±0.1
A_6-RNA*	5.4	25	0	3	39.0±0.1	88.2±1.1	255.9±3.5	11.9±0.1
A_6-RNA^{dA16*}	5.4	25	0	3	36.3±0.1	91.0±1.0	267.5±3.3	11.3±0.1
A_6-RNA^{m1rA16*}	5.4	25	0	3	26.5±0.3	61.2±2.7	177.5±8.9	8.2±0.1
A_6-RNA^{m1dA16*}	5.4	25	0	3	25.3±0.2	67.9±1.2	201.1±4.0	8.0±0.1
HIV-2 TAR	5.4	25	0	2.5	68.5±0.2	72.7±2.1	212.6±6.1	9.3±0.3
HIV-2 TAR^{m1rG26}	5.4	25	0	2.5	61.5±0.4	69.2±2.5	206.8±7.7	7.6±0.2
A_6-RNA	5.4	150	0	3	49.0±0.1	102.0±1.2	290.0±3.6	15.5±0.1
A_6-RNA^{dG10}	5.4	150	0	3	45.6±0.1	99.5±0.6	285.4±1.9	14.4±0.1
A_6-RNA^{m1rG10}	5.4	150	0	3	27.7±0.2	71.5±1.5	211.0±5.0	8.6±0.1
A_6-RNA^{m1dG10}	5.4	150	0	3	25.6± 0.2	64.5±3.0	189.2±9.9	8.0±0.1
A_6-RNA*	5.4	150	0	3	48.5±0.1	93.7±1.4	264.5±4.5	14.8±0.1
A_6-RNA^{dA16*}	5.4	150	0	3	45.6±0.1	98.2±2.6	281.6±8.1	14.3±0.2
A_6-RNA^{m1rA16*}	5.4	150	0	3	35.5±0.1	73.1±0.5	210.2±1.7	10.4±0.1
A_6-RNA^{m1dA16*}	5.4	150	0	3	34.0±0.1	75.8±0.3	220.2±0.9	10.2±0.1
A_6-RNA*	5.4	150	3	3	51.8±0.1	93.3±0.1	260.4±0.5	15.6±0.1
A_6-RNA^{dA16*}	5.4	150	3	3	49.5±0.1	97.6±1.4	276.0±4.4	15.4±0.1
A_6-RNA^{m1rA16*}	5.4	150	3	3	38.0±0.2	75.4±0.8	215.6±2.9	11.0±0.1
A_6-RNA^{m1dA16*}	5.4	150	3	3	36.2±0.3	76.0±1.6	219.2±5.0	10.7±0.1
A_6-RNA*	6.8	25	0	3	41.0±0.3	88.9±0.9	259.7±3.0	12.5±0.1
A_6-RNA^{dA16*}	6.8	25	0	3	38.2±0.2	95.2±1.0	279.1±3.3	12.0±0.1
A_6-RNA^{m1rA16*}	6.8	25	0	3	29.3±0.1	69.8±1.1	204.3±3.7	8.9±0.1
A_6-RNA^{m1dA16*}	6.8	25	0	3	27.3±0.1	72.6±1.2	214.9±3.9	8.5±0.1
A_6-RNA$^{*(K)}$	5.4	150	0	3	46.4±0.2	94.1±1.2	267.8±4.0	14.3±0.1
A_6-RNA$^{dA16*(K)}$	5.4	150	0	3	43.5±0.1	93.0±0.4	267.1±1.5	13.4±0.1
A_6-RNA$^{m1rA16*(K)}$	5.4	150	0	3	33.3±0.2	65.4±1.1	186.7±3.4	9.7±0.1

A_6-RNA$^{m1dA16*(K)}$	5.4	150	0	3	31.8±0.2	72.0±1.7	209.6±5.5	9.6±0.1
A_6-DNA	5.4	25	0	3	37.6±0.3	91.1±1.9	266.6±6.1	11.6±0.1
A_6-DNA^{rG10}	5.4	25	0	3	36.5±0.1	92.8±2.0	273.1±6.6	11.4±0.1
A_6-DNA^{m1rG10}	5.4	25	0	3	26.3±0.1	81.3±0.7	244.9±2.4	8.3±0.1
A_6-DNA^{m1dG10}	5.4	25	0	3	27.3±0.4	77.1±3.5	230.1±11.3	8.5±0.1
A_6-DNA^{rA16}	5.4	25	0	3	35.5±0.7	89.8±1.7	264.3±4.7	11.0±0.3
A_6-DNA^{m1rA16}	5.4	25	0	3	27.0±0.6	74.7±3.3	222.4±0.1	8.4±0.2
A_6-DNA^{m1dA16}	5.4	25	0	3	29.2±0.2	70.5±1.8	206.4±5.7	8.9±0.1
A_6-DNA$^{(K)}$	5.4	150	0	3	46.4±0.2	93.7±0.7	266.4±2.3	14.2±0.1
A_6-DNA$^{m1dA16(K)}$	5.4	150	0	3	37.9±0.1	72.5±0.4	206.4±1.0	11.0±0.1

Table S1. Thermodynamic parameters obtained from optical melting experiments on modified A_6-RNA and A_6-DNA duplexes, and HIV-2 TAR under various buffer conditions. C_t denotes the concentration of the double stranded/hairpin species at the start of the melting measurement, T_m is the melting temperature, while ΔH, ΔS and $\Delta G_{25°C}$ denote the enthalpy, entropy and free energy of the melting transition respectively. * denotes samples in which the single strands were purified using polyacrylamide gel electrophoresis (methods). (K) denotes samples for which the optical melting experiments were performed in a buffer containing 15 mM potassium phosphate, 150 mM potassium chloride, 0.1 mM EDTA at pH 5.4.

Construct	pH	[NaCl] /[KCl] (mM)	[$MgCl_2$] (mM)	C_t (μm)	-ΔT_m (°C)	$\Delta H_{syn\text{-}anti}$ (kcal/mol)	$\Delta S_{syn\text{-}anti}$ (cal/mol/ K)	$\Delta G_{syn\text{-}anti(25°C)}$ (kcal/mol)
A_2-DNA^{m1dGG10}	5.4	150	0	3	13.8±0.4	11.4±2.8	24.0±9.1	4.3±0.1
A_2-RNA^{m1rGG10}	5.4	150	0	3	16.1±0.1	16.1±1.8	34.4±5.8	5.8±0.1
gcDNA^{m1dGG4}	5.4	150	0	3	17.2±0.9	12.4±2.5	31.6±8.9	3.0±0.2
gcRNA^{m1rGG4}	5.4	150	0	3	26.9±0.6	27.3±2.9	68.5±8.9	7.0±0.2
gcDNA^{m1dGG4}	5.4	150	3	3	19.3±2.9	16.6±15.6	44.3±50.9	3.4±0.4
gcRNA^{m1rGG4}	5.4	150	3	3	24.6±1.0	22.5±10.5	53.5±32.3	6.7±0.9
A_2-DNA^{m1dGG10}	5.4	25	0	3	14.2±0.7	20.6±8.4	55.1±26.8	4.3±0.5
A_2-RNA^{m1rGG10}	5.4	25	0	3	16.8±0.3	25.1±5.7	62.5±17.9	6.4±0.4
A_2-DNA^{GG}	5.4	25	0	3	13.1±0.9	19.7±4.5	52.8±13.7	4.0±0.4
A_2-RNA^{GG}	5.4	25	0	3	15.4±0.3	22.9±4.6	57.1±13.9	5.9±0.4
A_2-DNA$^{GG(K)}$	5.4	150	0	3	10.2±0.7	14.1±7.1	33.0±21.4	4.3±0.7
A_2-RNA$^{GG(K)}$	5.4	150	0	3	15.3±0.1	21.0±4.6	49.5±14.4	6.2±0.3

Table S2. Thermodynamic parameters for flipping a purine base from *anti* to *syn* in DNA and RNA. Thermodynamic parameters for base flipping were estimated from optical melting measurements on m^1G-G/G-G mismatch containing duplexes and their C-G WC bp containing counterparts. C_t denotes the concentration of the duplex species at the start of melting measurements for both m^1G-G/G-G and C-G bp containing samples. ΔT_m denotes the change in melting temperature of the mismatched duplex relative to a duplex containing the C-G bp at the same position. $\Delta H_{syn\text{-}anti}$, $\Delta S_{syn\text{-}anti}$ and $\Delta G_{syn\text{-}anti(25°C)}$ denote the change in enthalpy, entropy and free energy accompanying base flipping. (K) denotes samples for which the optical melting experiments were performed in a buffer containing 15 mM potassium phosphate, 150 mM potassium chloride, 0.1 mM EDTA at pH 5.4.

Mismatch type	Number of occurrences		Number of occurrences in a canonical duplex context		Number of HG mismatches in a canonical duplex context	
**DNA/RNA**	**DNA**	**RNA**	**DNA**	**RNA**	**DNA**	**RNA**
G-G	114	684	9	30	7	29
A-A	121	2047	8	6	0	3
A-G	482	6996	31	20	26	4

Table S3. Summary of the statistics obtained from the survey of purine-purine mismatches in the PDB. “Canonical duplex context” refers to a bp that is surrounded by 2 WC bps on both sides.

Nucleic acid type	Mismatch type	PDB ID	*Syn* nucleotide	*Anti* nucleotide
DNA	G-G	1D80	B:21	A:4
DNA	G-G	1D80	A:9	B:16
DNA	G-G	3DPG	C:6	D:13
DNA	G-G	3DPG	D:6	C:13
DNA	G-G	4XZF	B:7	B:7
DNA	G-G	5DB9	T:13	P:4
DNA	G-G	5DBC	T:13	P:4
DNA	A-G	111D	B:21	A:4
DNA	A-G	111D	A:9	B:16
DNA	A-G	112D	B:21	A:4
DNA	A-G	112D	A:9	B:16
DNA	A-G	1DNM	A:4	B:21
DNA	A-G	1DNM	B:16	A:9
DNA	A-G	114D	B:21	A:4
DNA	A-G	114D	A:9	B:16
DNA	A-G	150D	B:21	A:4
DNA	A-G	150D	A:9	B:16
DNA	A-G	153D	A:3	B:22
DNA	A-G	153D	B:15	A:10
DNA	A-G	178D	B:21	A:4
DNA	A-G	178D	A:9	B:16
DNA	A-G	1D75	B:21	A:4
DNA	A-G	1D75	A:9	B:16
DNA	A-G	1D81	B:21	A:4
DNA	A-G	1D81	A:9	B:16
DNA	A-G	5DBB	T:13	P:4
DNA	A-G	1U4B	C:6	B:27
DNA	A-G	3CVS	E:8	F:18
DNA	A-G	3CVS	G:8	H:18
DNA	A-G	3CWT	F:18	E:8
DNA	A-G	3CWT	H:18	G:8
DNA	A-G	5DB8	T:13	P:4

DNA	A-G	5KN9	C:7	D:18
RNA	A-A	4J50	B:14	A:8
RNA	A-A	4J50	A:14	B:8
RNA	A-A	4YN6	A:14	B:8
RNA	A-G	2H1M	A:6	B:27
RNA	A-G	2H1M	B:22	A:11
RNA	A-G	420D	A:6	B:27
RNA	A-G	420D	B:22	A:11
RNA	G-G	2R1S	B:20	A:7
RNA	G-G	2R20	B:20	A:7
RNA	G-G	2R21	B:20	A:7
RNA	G-G	2R22	B:20	A:7
RNA	G-G	3CZW	X:8	X:11
RNA	G-G	3CZW	X:8	X:11
RNA	G-G	3D0M	X:8	X:11
RNA	G-G	3D0M	X:8	X:11
RNA	G-G	3R1C	B:6	A:3
RNA	G-G	3R1C	A:6	B:3
RNA	G-G	3R1D	A:6	B:6
RNA	G-G	3R1D	B:3	A:9
RNA	G-G	3R1D	A:3	B:9
RNA	G-G	3R1E	A:3	B:6
RNA	G-G	3R1E	B:3	A:6
RNA	G-G	3SJ2	A:8	B:14
RNA	G-G	3SJ2	A:11	B:11
RNA	G-G	3SJ2	B:8	A:14
RNA	G-G	4E5C	B:10	A:10
RNA	G-G	4KQ0	B:4	E:16
RNA	G-G	4KQ0	B:7	E:13
RNA	G-G	4KQ0	B:10	E:10
RNA	G-G	4KQ0	E:7	B:13
RNA	G-G	4KQ0	E:4	B:16
RNA	G-G	4KTG	B:204	E:216
RNA	G-G	4KTG	E:213	B:207

RNA	G-G	4KTG	E:210	B:210
RNA	G-G	4KTG	B:213	E:207
RNA	G-G	4KTG	E:204	B:216

Table S4. List of purine-purine (G-G/A-A/A-G) HG mismatches in DNA and RNA duplexes obtained from the survey of crystal structures in the PDB. A given nucleotide is specified by its chain ID and residue number.

PDB ID	Mismatch Sequence	Area$_{MM}$ Exo (Å^2)	Area$_{MM}$ NoExo (Å^2)	WC Sequence	Area$_{WC}$ Exo (Å^2)	Area$_{WC}$ NoExo (Å^2)	ΔArea Exo (Å^2)	ΔArea NoExo (Å^2)
1D80	TGG/CGA	16.65	3.93	TGG/CCA	12	2.37	4.65	1.56
1D80	TGG/CGA	17.52	4.65	TGG/CCA	12	2.37	5.52	2.28
3DPG	CGA/TGG	18.41	7.2	CGA/TCG	12.6	2.36	5.81	4.84
3DPG	CGA/TGG	16.14	4.7	CGA/TCG	12.6	2.36	3.54	2.34
4XZF	CGG/CGG	15.07	3.65	CGG/CCG	12.65	2.37	2.42	1.28
4XZF*	CGG/CGG	15.07	3.65	CGG/CCG	12.65	2.37	2.42	1.28
5DB9	TGA/TGA	14.17	3.33	TGA/TCA	11.95	2.36	2.22	0.97
5DBC	TGA/TGA	13.44	3.17	TGA/TCA	11.95	2.36	1.49	0.81
111D	TGG/CAA	16.19	5.44	TGG/CCA	12	2.37	4.19	3.07
111D	TGG/CAA	15.19	4.58	TGG/CCA	12	2.37	3.19	2.21
112D	TAG/CGA	15.43	5.32	TAG/CTA	12.26	2.24	3.17	3.08
112D	TAG/CGA	15.33	4.06	TAG/CTA	12.26	2.24	3.07	1.82
1DNM	CAA/TGG	15.71	6.07	CAA/TTG	12.49	2.23	3.22	3.84
1DNM	CAA/TGG	17.32	7.1	CAA/TTG	12.49	2.23	4.83	4.87
114D	TAG/CGA	17.9	6.87	TAG/CTA	12.26	2.24	5.64	4.63
114D	TAG/CGA	14.55	2.95	TAG/CTA	12.26	2.24	2.29	0.71
150D	TAG/CGA	15.6	3.56	TAG/CTA	12.26	2.24	3.34	1.32
150D	TAG/CGA	16	3.99	TAG/CTA	12.26	2.24	3.74	1.75
153D	GAG/CGC	11.05	2.75	GAG/CTC	18.24	4.6	-7.19	-1.85
153D	GAG/CGC	10.38	2.88	GAG/CTC	18.24	4.6	-7.86	-1.72
178D	TGG/CAA	14.13	5.45	TGG/CCA	12	2.37	2.13	3.08
178D	TGG/CAA	15.73	4.86	TGG/CCA	12	2.37	3.73	2.49
1D75	TAG/CGA	14.4	3.94	TAG/CTA	12.26	2.24	2.14	1.7
1D75	TAG/CGA	14.6	4.38	TAG/CTA	12.26	2.24	2.34	2.14
1D81	TGG/CAA	13.52	3.65	TGG/CCA	12	2.37	1.52	1.28
1D81	TGG/CAA	16.6	7.34	TGG/CCA	12	2.37	4.6	4.97
5DBB	TAA/TGA	13.26	4.31	TAA/TTA	12.36	2.23	0.9	2.08
1U4B	AGC/GAT	18.58	10.59	AGC/GCT	14.25	3.15	4.33	7.44
3CVS	AGT/AAT	19.42	9.26	AGT/ACT	15.84	3.11	3.58	6.15
3CVS	AGT/AAT	20.16	9.33	AGT/ACT	15.84	3.11	4.32	6.22

3CWT	AAT/AGT	21.54	12.1	AAT/ATT	17.56	3.05	3.98	9.05
3CWT	AAT/AGT	16.58	7.93	AAT/ATT	17.56	3.05	-0.98	4.88
5DB8	TAA/TGA	16.65	4.7	TAA/TTA	12.36	2.23	4.29	2.47
5KN9	CGG/CAG	18.59	6.48	CGG/CCG	12.65	2.37	5.94	4.11
2H1M	AGU/AAU	10.05	6.77	AGU/ACU	14.4	8.71	-4.35	-1.94
2H1M	AGU/AAU	11.97	7.95	AGU/ACU	14.4	8.71	-2.43	-0.76
420D	AGU/AAU	13.46	9.36	AGU/ACU	14.4	8.71	-0.94	0.65
420D	AGU/AAU	13.49	9.4	AGU/ACU	14.4	8.71	-0.91	0.69
2R1S	UGA/UGA	9.32	2.93	UGA/UCA	7.54	3.39	1.78	-0.46
2R20	UGA/UGA	9.38	2.98	UGA/UCA	7.54	3.39	1.84	-0.41
2R21	UGA/UGA	9.55	3.07	UGA/UCA	7.54	3.39	2.01	-0.32
2R22	UGA/UGA	9.57	3.26	UGA/UCA	7.54	3.39	2.03	-0.13
3CZW	UGA/UGA	8.94	2.6	UGA/UCA	7.54	3.39	1.4	-0.79
3CZW	UGA/UGA	8.94	2.6	UGA/UCA	7.54	3.39	1.4	-0.79
3D0M	UGA/UGA	8.01	2.22	UGA/UCA	7.54	3.39	0.47	-1.17
3D0M	UGA/UGA	8.01	2.22	UGA/UCA	7.54	3.39	0.47	-1.17
3R1C	CGG/CGG	8.67	2.21	CGG/CCG	9.23	3.48	-0.56	-1.27
3R1C	CGG/CGG	9.13	2.26	CGG/CCG	9.23	3.48	-0.1	-1.22
3R1D	CGG/CGG	8.92	2.35	CGG/CCG	9.23	3.48	-0.31	-1.13
3R1D	CGG/CGG	7.96	1.57	CGG/CCG	9.23	3.48	-1.27	-1.91
3R1D*	CGG/CGG	8.75	2.33	CGG/CCG	9.23	3.48	-0.48	-1.15
3R1D*	CGG/CGG	9.73	2.98	CGG/CCG	9.23	3.48	0.5	-0.5
3R1D	CGG/CGG	8.99	2.21	CGG/CCG	9.23	3.48	-0.24	-1.27
3R1E	CGG/CGG	10.33	2.76	CGG/CCG	9.23	3.48	1.1	-0.72
3R1E	CGG/CGG	8.91	2.76	CGG/CCG	9.23	3.48	-0.32	-0.72
3R1E*	CGG/CGG	9.05	2.57	CGG/CCG	9.23	3.48	-0.18	-0.91
3SJ2	CGG/CGG	8.53	2.22	CGG/CCG	9.23	3.48	-0.7	-1.26
3SJ2	CGG/CGG	8.35	2.13	CGG/CCG	9.23	3.48	-0.88	-1.35
3SJ2	CGG/CGG	8.35	2.15	CGG/CCG	9.23	3.48	-0.88	-1.33
4E5C	CGG/CGG	8.6	2.35	CGG/CCG	9.23	3.48	-0.63	-1.13
4KQ0	CGG/CGG	8.92	1.65	CGG/CCG	9.23	3.48	-0.31	-1.83
4KQ0	CGG/CGG	8.71	2.1	CGG/CCG	9.23	3.48	-0.52	-1.38
4KQ0	CGG/CGG	8.95	2.27	CGG/CCG	9.23	3.48	-0.28	-1.21
4KQ0	CGG/CGG	8.69	2.1	CGG/CCG	9.23	3.48	-0.54	-1.38

4KQ0	CGG/CGG	8.9	1.64	CGG/CCG	9.23	3.48	-0.33	-1.84
4KTG	GGC/GGC	14.91	7.73	GGC/GCC	17.51	8.93	-2.6	-1.2
4KTG	GGC/GGC	18.72	10.06	GGC/GCC	17.51	8.93	1.21	1.13
4KTG	GGC/GGC	17.9	9.39	GGC/GCC	17.51	8.93	0.39	0.46
4KTG	GGC/GGC	18.66	9.99	GGC/GCC	17.51	8.93	1.15	1.06
4KTG	GGC/GGC	14.94	7.76	GGC/GCC	17.51	8.93	-2.57	-1.17

Table S5. Changes in stacking interactions (relative to WC bps) accompanying the formation of *syn-anti* G-G and G-A mismatches obtained from a survey of crystal structures in the PDB. PDB IDs marked with a * denote entries corresponding to multiple conformations of a given mismatch. "Mismatch Sequence" refers to the sequence of triplet of base pairs consisting of the mismatch (*syn* base underlined) and its immediate neighbors specified in a 5′ to 3′ direction. For example, TGG/CGA corresponds to 5′-TG(*syn*)G-3′/5′-CG(*anti*)A-3′. Area$_{MM}$ Exo and Area$_{MM}$ NoExo denote the stacking overlap area between the mismatch and its immediate neighbors computed using 3DNA (7) (see 'Materials and Methods' section), with and without the inclusion of exocyclic groups. "WC Sequence" denotes the sequence of the idealized WC base paired duplex constructed using the sequence of the mismatched strand containing the *syn* base, specified in a 5′ to 3′ direction. For example, the WC base paired duplex corresponding to TGG/CGA would be 5′-TGG-3′/5′-CCA-3′ or TGG/CCA. Area$_{WC}$ Exo and Area$_{WC}$ NoExo denote the stacking overlap area between the central WC bp and its immediate neighbors. ΔArea Exo (Area$_{MM}$ Exo-Area$_{WC}$ Exo) and ΔArea NoExo (Area$_{MM}$ NoExo-Area$_{WC}$ NoExo) denote the change in overlap area between the mispaired and WC base paired triplet of base pairs.

Base pair	0 < χ < 90°	C1′- C1′ distance < 9.5 Å	H-bond distances < 3.5 Å	DNA HG	DNA HG*	RNA HG	RNA HG*
	N	N	N	0.00 (0.00)	0.00 (0.00)	0.00 (0.00)	0.00 (0.00)
A_6-DNA	N	N	Y	0.00 (0.00)	0.00 (0.00)	0.00 (0.00)	0.00 (0.00)
A16-T9	N	Y	N	0.00 (0.00)	0.00 (0.00)	0.00 (0.00)	0.00 (0.00)
(bsc0)	N	Y	Y	0.00 (0.00)	0.06 (0.06)	0.00 (0.00)	0.00 (0.00)
/	Y	N	N	0.01 (0.01)	0.00 (0.01)	0.78 (0.78)	0.40 (0.41)
A_6-RNA	Y	N	Y	0.02 (0.02)	0.02 (0.02)	0.01 (0.01)	0.04 (0.03)
A16-U9	Y	Y	N	0.02 (0.05)	0.01 (0.04)	0.00 (0.01)	0.01 (0.07)
(OL3)	Y	Y	Y	0.95 (0.92)	0.90 (0.87)	0.20 (0.19)	0.54 (0.49)
	$\Delta G_{constrict}$ (kcal/mol)			-3.02 (-2.91)	-3.17 (-3.04)	0.81 (0.83)	-0.17 (-0.10)
	$\Delta\Delta G_{constrict}$ (kcal/mol)				3.42 (3.34)		
	N	N	N	0.00 (0.00)	0.00 (0.00)	0.00 (0.00)	0.00 (0.00)
A_6-DNA	N	N	Y	0.00 (0.00)	0.00 (0.00)	0.00 (0.00)	0.00 (0.00)
G10-C15	N	Y	N	0.00 (0.00)	0.00 (0.00)	0.00 (0.00)	0.00 (0.00)
(bsc0)	N	Y	Y	0.01 (0.01)	0.00 (0.00)	0.00 (0.00)	0.00 (0.00)
/	Y	N	N	0.04 (0.04)	0.04 (0.04)	0.94 (0.94)	0.97 (0.97)
A_6-RNA	Y	N	Y	0.01 (0.01)	0.01 (0.01)	0.00 (0.00)	0.00 (0.00)
G10-C15	Y	Y	N	0.02 (0.10)	0.03 (0.10)	0.01 (0.02)	0.01 (0.01)
(OL3)	Y	Y	Y	0.91 (0.83)	0.93 (0.85)	0.04 (0.04)	0.02 (0.01)
	$\Delta G_{constrict}$ (kcal/mol)			-1.85 (-1.78)	-1.87 (-1.81)	1.83 (1.95)	2.41 (2.50)
	$\Delta\Delta G_{constrict}$ (kcal/mol)				3.98 (4.02)		
A_2-DNA	N	N	N	0.00 (0.00)	0.00 (0.00)	0.00 (0.00)	0.04 (0.04)
A16-T9	N	N	Y	0.00 (0.00)	0.00 (0.00)	0.00 (0.00)	0.00 (0.00)
(bsc0)	N	Y	N	0.00 (0.00)	0.00 (0.00)	0.00 (0.00)	0.00 (0.00)
/	N	Y	Y	0.00 (0.00)	0.00 (0.00)	0.00 (0.00)	0.00 (0.00)
A_2-RNA	Y	N	N	0.01 (0.01)	0.01 (0.01)	0.53 (0.53)	0.58 (0.58)
A16-U9	Y	N	Y	0.03 (0.03)	0.03 (0.03)	0.04 (0.04)	0.04 (0.04)
(OL3)	Y	Y	N	0.02 (0.08)	0.02 (0.08)	0.01 (0.02)	0.00 (0.01)

	Y	Y	Y	0.94 (0.88)	0.94 (0.88)	0.42 (0.40)	0.34 (0.33)
	$\Delta G_{constrict}$ (kcal/mol)			-2.81 (-2.64)	-2.83 (-2.64)	0.14 (0.16)	0.32 (0.34)
	$\Delta\Delta G_{constrict}$ (kcal/mol)			3.05 (2.89)			
	N	N	N	0.00 (0.00)	0.01 (0.01)	-	-
	N	N	Y	0.00 (0.00)	0.00 (0.00)	-	-
	N	Y	N	0.00 (0.00)	0.00 (0.00)	-	-
A_6-DNA	N	Y	Y	0.01 (0.01)	0.05 (0.05)	-	-
A16-T9	Y	N	N	0.04 (0.05)	0.02 (0.02)	-	-
(bsc1)	Y	N	Y	0.03 (0.03)	0.03 (0.03)	-	-
	Y	Y	N	0.01 (0.03)	0.01 (0.03)	-	-
	Y	Y	Y	0.89 (0.88)	0.89 (0.87)	-	-
	$\Delta G_{constrict}$ (kcal/mol)			-1.78 (-1.75)	-2.24 (-2.20)	-	-
	N	N	N	0.00 (0.00)	0.00 (0.00)	-	-
	N	N	Y	0.00 (0.00)	0.00 (0.00)	-	-
	N	Y	N	0.00 (0.00)	0.00 (0.00)	-	-
A_6-DNA	N	Y	Y	0.00 (0.00)	0.00 (0.00)	-	-
A16-T9	Y	N	N	0.01 (0.01)	0.00 (0.00)	-	-
(OL15)	Y	N	Y	0.01 (0.01)	0.01 (0.01)	-	-
	Y	Y	N	0.02 (0.04)	0.02 (0.04)	-	-
	Y	Y	Y	0.97 (0.95)	0.97 (0.95)	-	-
	$\Delta G_{constrict}$ (kcal/mol)			-3.11 (-3.06)	-3.48 (-3.41)	-	-

Table S6. Fractional populations of conformational states of the A16(*syn*)-T/U9 and G10(*syn*)-C15$^+$ HG bps in MD simulations of A_6 and A_2 DNA and RNA. The base pairing geometries were characterized using the following geometric criteria - a C1′-C1′ distance cutoff of 9.5 Å, hydrogen bond donor-acceptor distance of 3.5 Å and a purine χ angle between 0° and 90°. Y/N denotes whether the given geometric criterion is satisfied or not. A HG bp was considered to be formed only when the donor-acceptor

distances for both the constituent hydrogen bonds were less than the cutoff. HG and HG* refer to the starting geometry of the A16(*syn*)-T/U9 and G10(*syn*)-$C15^+$ bps (see 'Materials and Methods') in the simulations. The energetic cost for constricting the bases ($\Delta G_{constrict}$) in a given simulation was defined as the negative logarithm of the ratio of the population of the constricted HG bp (p_{YYY}) to that of the HG* bp (p_{YNN}) that is not constricted i.e., $\Delta G_{constrict} = -RT \ln(p_{YYY} / p_{YNN})$, where R denotes the universal gas constant and T the temperature (25 °C). The energetic cost for constricting the bases in a given system for a particular force field, say A_6-DNA for the bsc0 force field was defined as the average of $\Delta G_{constrict}$ over the two simulation setups with HG and HG* starting geometries. For example, $\Delta G_{constrict(A6\text{-}DNA,\ bsc0)} = 0.5 * (\Delta G_{constrict(A6\text{-}DNA,\ bsc0,\ HG)} + \Delta G_{constrict(A6\text{-}DNA,\ bsc0,\ HG*)})$. $\Delta\Delta G_{constrict}$ is defined as the additional energetic cost to constrict the bases in RNA relative to DNA, for a given pair of DNA/RNA systems/force fields i.e., $\Delta\Delta G_{constrict} = \Delta G_{constrict(A6\text{-}RNA,\ OL3)} - \Delta G_{constrict(A6\text{-}DNA,\ bsc0)}$. For example, the extra energetic cost to constrict the bases in A_6-RNA (OL3) relative to A_6-DNA (bsc0) is given by 0.3 – (-3.1) = 3.4 kcal/mol. The obtained populations and energies were also seen to be robust to the inclusion of a hydrogen-donor-acceptor angle cutoff of < 30° to additionally define the formation of a hydrogen bond (values in parentheses).

System	Force field	Base pair	Starting geometry	C1′-C1′ Distance (Å)	Purine Χ (°)	HG hbond (Å)	Other hbond (Å)
A_6-DNA	bsc0	A16(*syn*)-T9	HG	8.90±0.31	39.43±11.39	3.00±0.17	2.96±0.24
A_6-DNA	bsc0	A16(*syn*)-T9	HG*	8.89±0.31	34.94±18.04	3.00±0.14	2.95±0.19
A_6-DNA	bsc1	A16(*syn*)-T9	HG	9.00±0.40	62.26±11.79	3.16±0.56	3.00±0.44
A_6-DNA	bsc1	A16(*syn*)-T9	HG*	8.97±0.44	64.46±14.65	3.09±0.38	2.96±0.41
A_6-DNA	OL15	A16(*syn*)-T9	HG	8.81±0.32	51.81±10.12	2.99±0.17	2.97±0.21
A_6-DNA	OL15	A16(*syn*)-T9	HG*	8.80±0.29	52.04±10.18	2.99±0.14	2.97±0.21
A_2-DNA	bsc0	A16(*syn*)-T9	HG	8.96±0.33	38.47±11.27	3.01±0.17	2.96±0.19
A_2-DNA	bsc0	A16(*syn*)-T9	HG*	8.97±0.30	38.30±11.30	3.01±0.20	2.96±0.23
A_6-DNA	bsc0	G10(*syn*)-$C15^+$	HG	8.90±0.39	37.39±14.33	3.04±0.32	2.88±0.21
A_6-DNA	bsc0	G10(*syn*)-$C15^+$	HG*	8.89±0.38	38.94±13.03	3.03±0.30	2.87±0.18
A_6-RNA	OL3	A16(*syn*)-U9	HG	11.56±1.63	44.16±12.81	5.33±1.61	4.03±1.66
A_6-RNA	OL3	A16(*syn*)-U9	HG*	10.09±1.51	41.70±13.15	3.91±1.17	3.07±0.54
A_2-RNA	OL3	A16(*syn*)-U9	HG	10.51±1.50	43.56±13.46	4.21±1.22	3.10±0.66
A_2-RNA	OL3	A16(*syn*)-U9	HG*	10.77±1.45	49.55±19.70	4.34±1.11	3.09±0.53
A_6-RNA	OL3	G10(*syn*)-$C15^+$	HG	10.72±0.64	38.05±10.98	4.96±0.61	3.28±0.49
A_6-RNA	OL3	G10(*syn*)-$C15^+$	HG*	10.80±0.55	38.18±10.86	5.02±0.53	3.28±0.50

Table S7. Geometric characteristics of HG bps in MD simulations of A_2 and A_6 DNA and RNA. Average values and standard deviations of geometric criteria defining the formation of a HG bp – C1′- C1′ distance, purine χ angle, HG hydrogen bond (A(N7)-T/U(N3) or G(N7)– C^+(N3)) and other hydrogen bond (A(N6)-T/U(O4) or G(O6)-C^+(N4)) in MD simulations of A_6/A_2 DNA and RNA duplexes with different starting geometries (see 'Materials and Methods' section) and force fields.

References

1. Zhou, H., Kimsey, I.J., Nikolova, E.N., Sathyamoorthy, B., Grazioli, G., McSally, J., Bai, T., Wunderlich, C.H., Kreutz, C., Andricioaei, I. et al. (2016) m^1A and m^1G disrupt A-RNA structure through the intrinsic instability of Hoogsteen base pairs. Nat. Struct. Mol. Biol., 23, 803-810.
2. Nikolova, E.N., Kim, E., Wise, A.A., O'Brien, P.J., Andricioaei, I. and Al-Hashimi, H.M. (2011) Transient Hoogsteen base pairs in canonical duplex DNA. Nature, 470, 498-502.
3. Fonville, J.M., Swart, M., Vokáčová, Z., Sychrovský, V., Šponer, J.E., Šponer, J., Hilbers, C.W., Bickelhaupt, F.M. and Wijmenga, S.S. (2012) Chemical shifts in nucleic acids studied by density functional theory calculations and comparison with experiment. Chem. - Eur. J. , 18, 12372-12387.
4. Merriman, D.K., Xue, Y., Yang, S., Kimsey, I.J., Shakya, A., Clay, M. and Al-Hashimi, H.M. (2016) Shortening the HIV-1 TAR RNA bulge by a single nucleotide preserves motional modes over a broad range of time scales. Biochemistry, 55, 4445-4456.
5. Yildirim, I., Park, H., Disney, M.D. and Schatz, G.C. (2013) A dynamic structural model of expanded RNA CAG repeats: A refined X-ray structure and computational investigations using molecular dynamics and umbrella sampling simulations. J. Am. Chem. Soc., 135, 3528-3538.
6. Le Novère, N. (2001) MELTING, computing the melting temperature of nucleic acid duplex. Bioinformatics, 17, 1226-1227.
7. Lu, X. and Olson, W.K. (2003) 3DNA: a software package for the analysis, rebuilding and visualization of three-dimensional nucleic acid structures. Nucleic Acids Res. , 31, 5108-5121.
8. Sathyamoorthy, B., Shi, H., Zhou, H., Xue, Y., Rangadurai, A., Merriman, D.K. and Al-Hashimi, H.M. (2017) Insights into Watson-Crick/Hoogsteen breathing dynamics and damage repair from the solution structure and dynamic ensemble of DNA duplexes containing m^1A. Nucleic Acids Res. , 45, 5586-5601.
9. Shi, H., Clay, M.C., Rangadurai, A., Sathyamoorthy, B., Case, D.A. and Al-Hashimi, H.M. (2018) Atomic structures of excited state A-T Hoogsteen base pairs in duplex DNA by combining NMR relaxation dispersion, mutagenesis, and chemical shift calculations. J. Biomol. NMR, 70, 229-244.
10. Zhou, H., Hintze, B.J., Kimsey, I.J., Sathyamoorthy, B., Yang, S., Richardson, J.S. and Al-Hashimi, H.M. (2015) New insights into Hoogsteen base pairs in DNA duplexes from a structure-based survey. Nucleic Acids Res., 43, 3420-3433.
